# Nanoscale imaging of Fe-rich inclusions in single-crystal zircon using X-ray ptycho-tomography

**DOI:** 10.1038/s41598-024-55846-4

**Published:** 2024-03-01

**Authors:** Venkata S. C. Kuppili, Matthew Ball, Darren Batey, Kathryn Dodds, Silvia Cipiccia, Kaz Wanelik, Roger Fu, Christoph Rau, Richard J. Harrison

**Affiliations:** 1 Department of Earth Sciences, University of Cambridge, Downing Street, Cambridge, CB2 3EQ UK; 2 Diamond Light Source, Harwell Campus, Didcot, OX11 0DE UK; 3 Department of Earth and Planetary Sciences, Harvard University, Cambridge, MA 02138 USA; 4 Canadian Light Source, University of Saskatchewan, 44 Innovation Boulevard, Saskatoon, SK S7N 2V3 Canada; 5 Department of Medical Physics and Biomedical Engineering, University College London, Gower St, London, WC1E 6BT UK

**Keywords:** Planetary science, Solid Earth sciences, Astronomy and planetary science, Physics

## Abstract

We apply X-ray ptycho-tomography to perform high-resolution, non-destructive, three-dimensional (3D) imaging of Fe-rich inclusions in paleomagnetically relevant materials (zircon single crystals from the Bishop Tuff ignimbrite). Correlative imaging using quantum diamond magnetic microscopy combined with X-ray fluorescence mapping was used to locate regions containing potential ferromagnetic remanence carriers. Ptycho-tomographic reconstructions with voxel sizes 85 nm and 21 nm were achievable across a field-of-view > 80 µm; voxel sizes as small as 5 nm were achievable over a limited field-of-view using local ptycho-tomography. Fe-rich inclusions 300 nm in size were clearly resolved. We estimate that particles as small as 100 nm—approaching single-domain threshold for magnetite—could be resolvable using this “dual-mode” methodology. Fe-rich inclusions (likely magnetite) are closely associated with apatite inclusions that have no visible connection to the exterior surface of the zircon (e.g., via intersecting cracks). There is no evidence of radiation damage, alteration, recrystallisation or deformation in the host zircon or apatite that could provide alternative pathways for Fe infiltration, indicating that magnetite and apatite grew separately as primary phases in the magma, that magnetite adhered to the surfaces of the apatite, and that the magnetite-coated apatite was then encapsulated as primary inclusions within the growing zircon. Rarer examples of Fe-rich inclusions entirely encapsulated by zircon are also observed. These observations support the presence of primary inclusions in relatively young and pristine zircon crystals. Combining magnetic and tomography results we deduce the presence of magnetic carriers that are in the optimal size range for carrying strong and stable paleomagnetic signals but that remain below the detection limits of even the highest-resolution X-ray tomography reconstructions. We recommend the use of focused ion beam nanotomography and/or correlative transmission electron microscopy to directly confirm the presence of primary magnetite in the sub 300 nm range as a necessary step in targeted paleomagnetic workflows.

## Introduction

Magnetic minerals are ubiquitous in the natural environment. These minerals retain a memory of the geomagnetic field that was present during the rock’s formation, yielding paleomagnetic information that can be used to track the movements of tectonic plates and reveal the history Earth’s geodynamo. Extracting reliable paleomagnetic information from bulk rocks becomes progressively harder with increasing age: the older the rock, the more complex is its geological history, and the more likely it is to have experienced conditions (e.g., metamorphic heating or exposure to geothermal fluids) that altered, or even destroyed, its primary magnetic information. To overcome the inherent problems with bulk-rock measurements, it becomes necessary to make observations at ever decreasing length scales, allowing paleomagnetic measurements to be targeted at specific regions-of-interest (ROI) that contain primary remanence carriers with ideal recording properties and in sufficient numbers to deliver a high-fidelity record of the ancient magnetic field (e.g.,^1^). One successful approach to solving this problem takes advantage of ultrasensitive 3-component DC superconducting quantum interference device (SQUID) magnetometers to target individual single crystals of silicate minerals (e.g. quartz, feldspar, zircon) extracted from the bulk rock, each containing multiple inclusions of magnetic minerals, such as magnetite^2,3^. The small size of the magnetite inclusions (which imparts optimal magnetic recording properties) and their encapsulation within a silicate host (which provides protection from chemical alteration) means they have a good chance of carrying a primary magnetic signal. The single-crystal paleomagnetic method is one of several highly targeted approaches [e.g.,^4,5^] designed to circumvent the confounding signals that bedevil bulk paleomagnetic measurements, such as those associated with secondary or less ideal remanence carriers, with particular relevance to studies of ancient terrestrial and extraterrestrial samples [e.g.,^6–15^].

**Figure 1:**
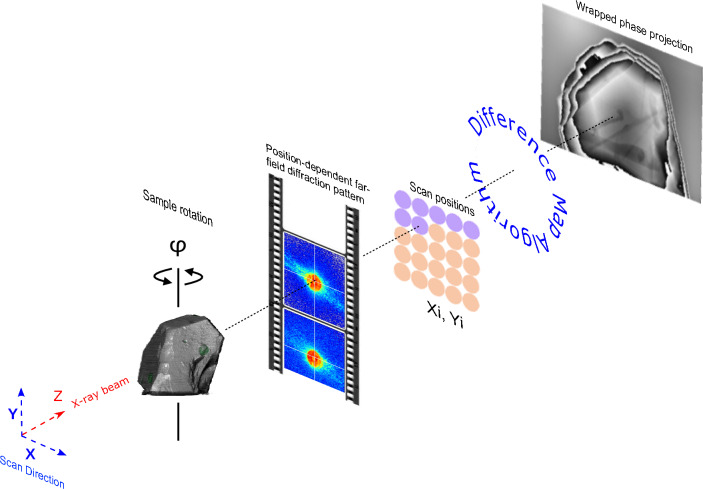
Schematic of X-ray ptycho-tomography experimental setup. A sample at a given angle φ is scanned perpendicular to the X-ray propagation direction, collecting far-field diffraction patterns at well known positions Xi, Yi. These patterns, along with the information about the positions at which they were collected, are processed using a phase retrieval (Difference Map) algorithm, thus obtaining high-resolution 2D complex transmission projections of the sample at the given angle, along with the illumination function incident on the sample at this angle. These projections, after relevant post processing steps, are combined using tomography obtaining a high-resolution (10 s of nm) 3D map of the sample.

Shrinking the length scale of paleomagnetic observations means that understanding the magnetic content of the ROI becomes increasingly important. Three-dimensional (3D), ‘non-destructive’ imaging (i.e., imaging that preserves both the physical integrity of the sample *and* the paleomagnetic signals it carries) is helpful to assess the origin and fidelity of the magnetic information carried. However, the 3D imaging requirements are highly demanding. The shapes of individual particles should ideally be defined with resolution approaching the micromagnetic magnetic exchange length (~ 3 nm for metallic Fe and ~ 10 nm for magnetite;^16^) so that finite-element micromagnetic simulations can be used to predict how morphological features control a particle’s magnetic properties. Particle sizes of stable remanence carriers range from 10 to 1000 s of nm, spanning the single-domain (SD) to single-vortex (SV) to multi-vortex (MV) range of micromagnetic behavior^16^. Single crystals can extend over several hundred µm, requiring high spatial resolution over a wide field-of-view (FOV). The present study aims solve the problem of non-destructive imaging of magnetic inclusions within dense zircon single crystals. To do this we use the technique of X-ray ptycho-tomography (Fig. 1), which combines X-ray ptychography with conventional tomographic reconstruction^17^. Ptychography is a scanning coherent diffraction imaging (CDI) technique that reconstructs the two-dimensional (2D) complex transmission function of the sample along with the incident X-ray probe function from far-field diffraction patterns obtained at well known, pre-defined scan points on the sample^18–21^. The scan-points are chosen such that there is overlap between the adjacent points. The overlap ensures that the ptychographic imaging problem is over-determined. Along with sufficient overlap, the diffraction patterns need to be ‘over-sampled’ to obtain optimum digital representation of the analog exit wave in the far-field regime^22^. Sufficient overlap in real space along with optimum over-sampling in inverse space guarantee the feasibility of ptychographic imaging. Spatial resolution depends, however, on a number of experimental and computational factors, including the phase contrast between the minerals being investigated.

We apply a dual-mode X-ray ptycho-tomography approach (see section “X-ray ptycho-tomography and X-ray fluorescence measurements”) to study the internal microstructure of single crystals of the mineral zircon (ZrSiO_4_), including a survey (S) mode that yields large FOV with low to moderate resolution and a high-resolution (HR) mode that yields high resolution over a targeted FOV. We focus on two zircons crystals (RF1 and RF2) separated from bulk samples of the 767.1 ka Bishop Tuff, California (see section “Sample selection and initial characterization”). The paleomagnetism of the Bishop Tuff has been studied previously using both conventional bulk paleomagnetic methods^23,24^ and the zircon single-crystal paleomagnetic method^25^. The study of Fu et al.^25^ serves as a proof-of-concept for the zircon single-crystal paleomagnetic method, demonstrating mean paleointensity results consistent with those of bulk methods (albeit with a greater standard deviation due to the smaller sample size). Only ferromagnetic inclusions (e.g., magnetite) contribute to the paleomagnetism of the samples. We therefore correlate chemical and magnetic auxiliary information with the structural information obtained through ptycho-tomography to identify Fe-rich inclusions most likely to be magnetite.

## Results

### Chemical composition and inclusion mineralogy

Chemical mapping shows that the major elements in both RF1 and RF2 are Zr, Si and O, associated with the host zircon crystal, and Ca associated with the dominant inclusion phase of apatite (Fig. S2). Separate peaks for Zr L_α_ at 2.042 keV and P K_α_ at 2.013 keV were not resolvable, but the presence of Zr was confirmed by observing the Zr K_α_ peak at 15.744 keV in the 30 kV data of RF2. Minor elements such as Na, K and Al are associated with likely inclusions of alkali feldspar visible within the apatite in Fig. S2b. Fe-rich inclusions are visible on the surface of the zircon crystals, in cracks, and associated with apatite (as previously observed by Fu et al.^25^). A significant signal at 8.63 keV, corresponding to Zn K_α_ is associated with the very bright feature observed at the surface in Fig. S2c, and is likely surface contamination.

### External morphology

**Figure 2:**
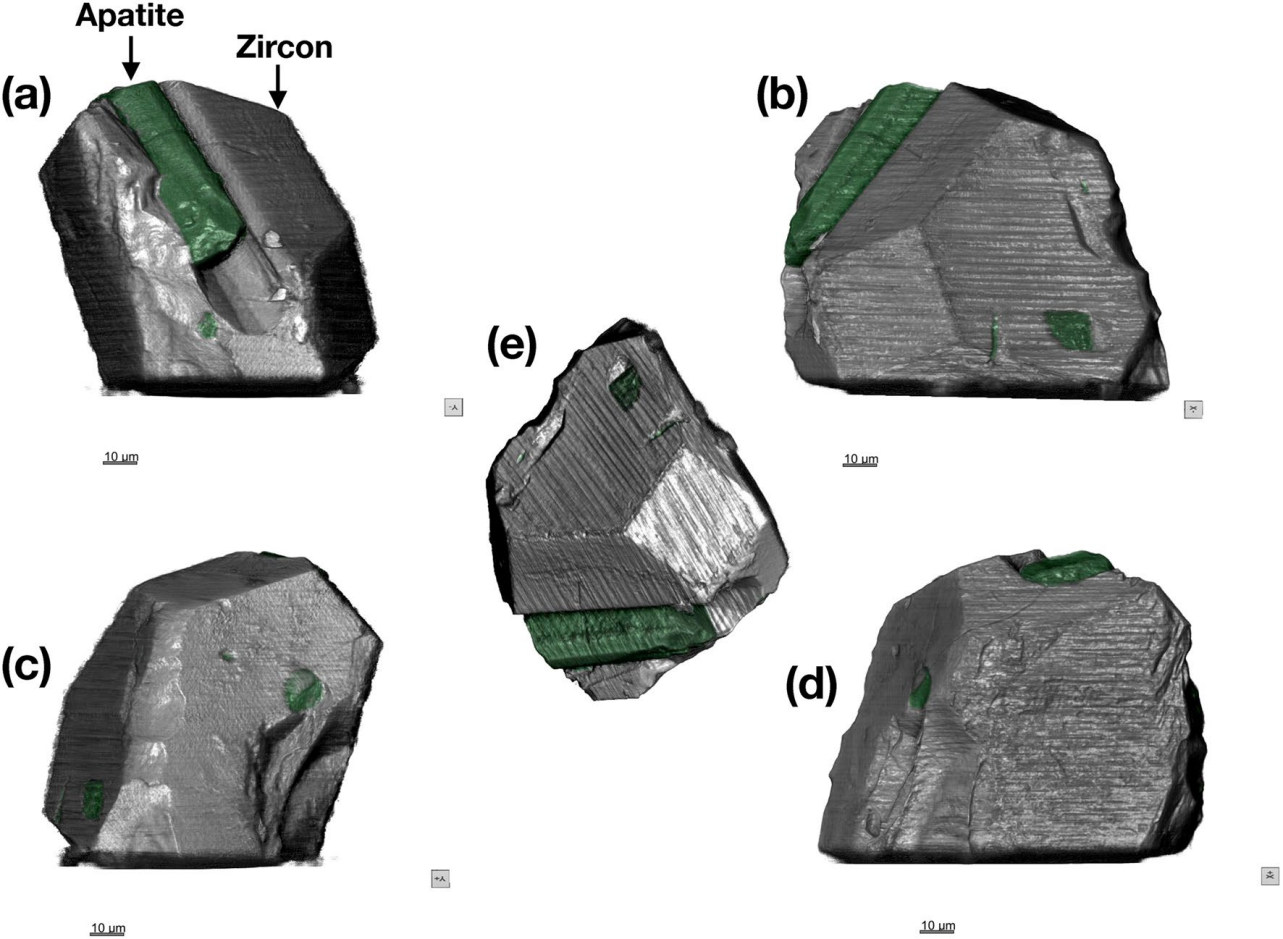
External morphology of crystal RF1. (**a**–**e**) 85 × 85 × 85 nm voxel size phase reconstruction. Apatite inclusions are highlighted in green. Images in (**a**–**d**) are related by 90° rotation. The image orientation in (**e**) can be compared with the SE image and EDX chemical map in Fig. S2a-b.

**Figure 3:**
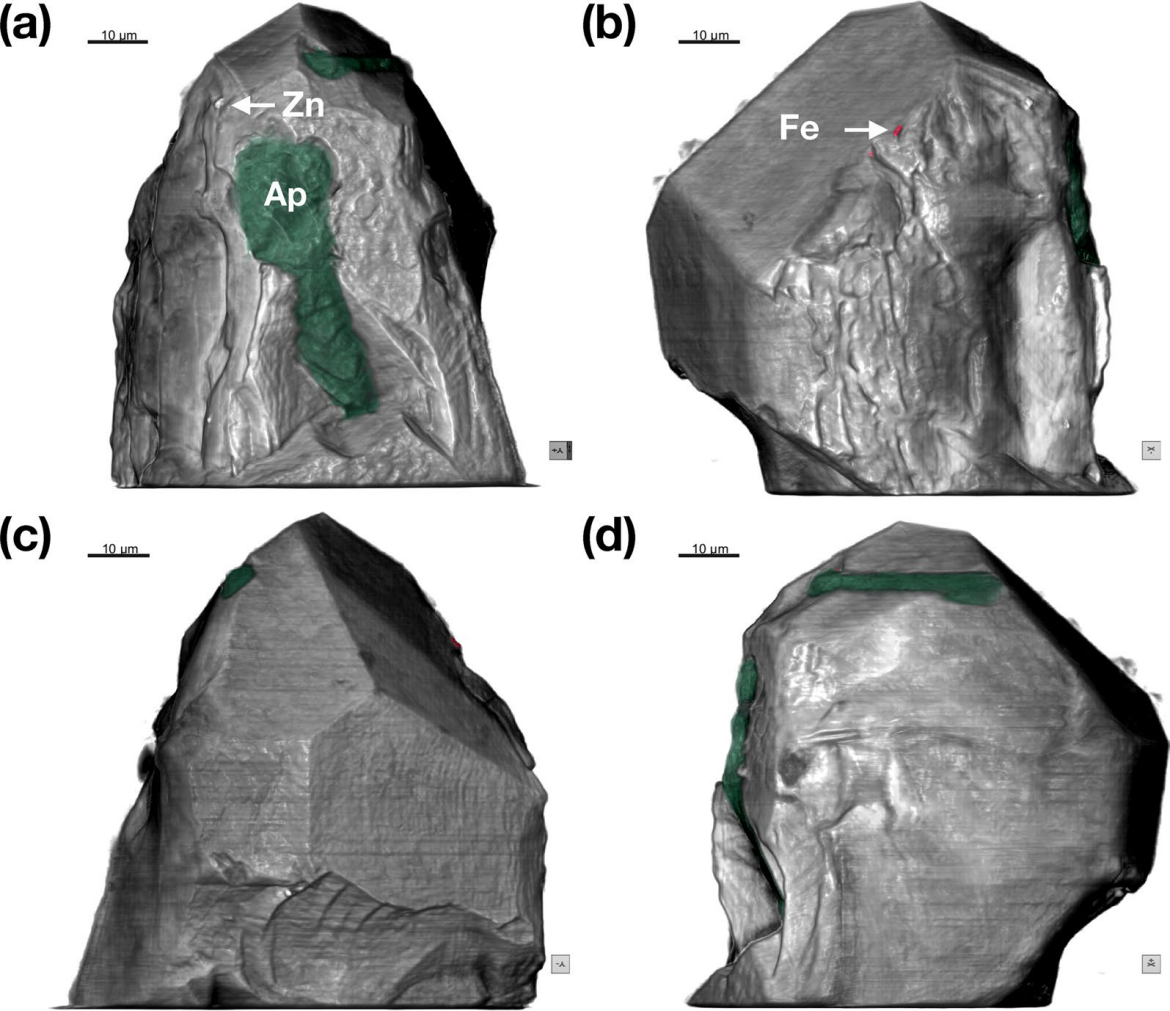
External Morphology of crystal RF2. (**a**–**d**) 85 × 85 × 85 nm voxel size phase reconstruction. Apatite inclusions are highlighted in green, Fe-rich particles are in red, Zn refers to the Zn-bearing particle highlighted in Fig. S2c. Images in (**a**–**d**) are related by 90° rotation. The image in (**a**) can be compared with the SE image and EDX chemical map in Fig. S2c-d.

Surface visualizations of the 85 nm voxel size reconstructions for RF1 and RF2 are shown in in Figs. 2 and 3, respectively. A partial surface of RF2 using the 20 nm reconstruction is shown in Fig. S4. The rich detail of these reconstructions can be compared directly with the SE images presented in Fig. S2 and demonstrates that the quality and spatial resolution of the ptycho-tomographic imaging compares favourably to that obtainable by scanning electron microscopy at comparable magnifications. An apatite crystal with a hexagonal prismatic habit is clearly visible in Fig. 2a, as well as the hexagonal impression in the zircon host left behind when part of this apatite crystal fractured and fell out. The bright Zn-bearing particle on the surface of RF2, highlighted in Fig. S2c, is clearly resolved in Fig. 3a, along with rich details of the zircon’s surface features, including cracks, pits, facets, and fractures. A 2.3 µm long Fe-rich particle is highlighted at the surface of RF2 in Fig. 3b.

### Internal distribution of inclusions

**Figure 4:**
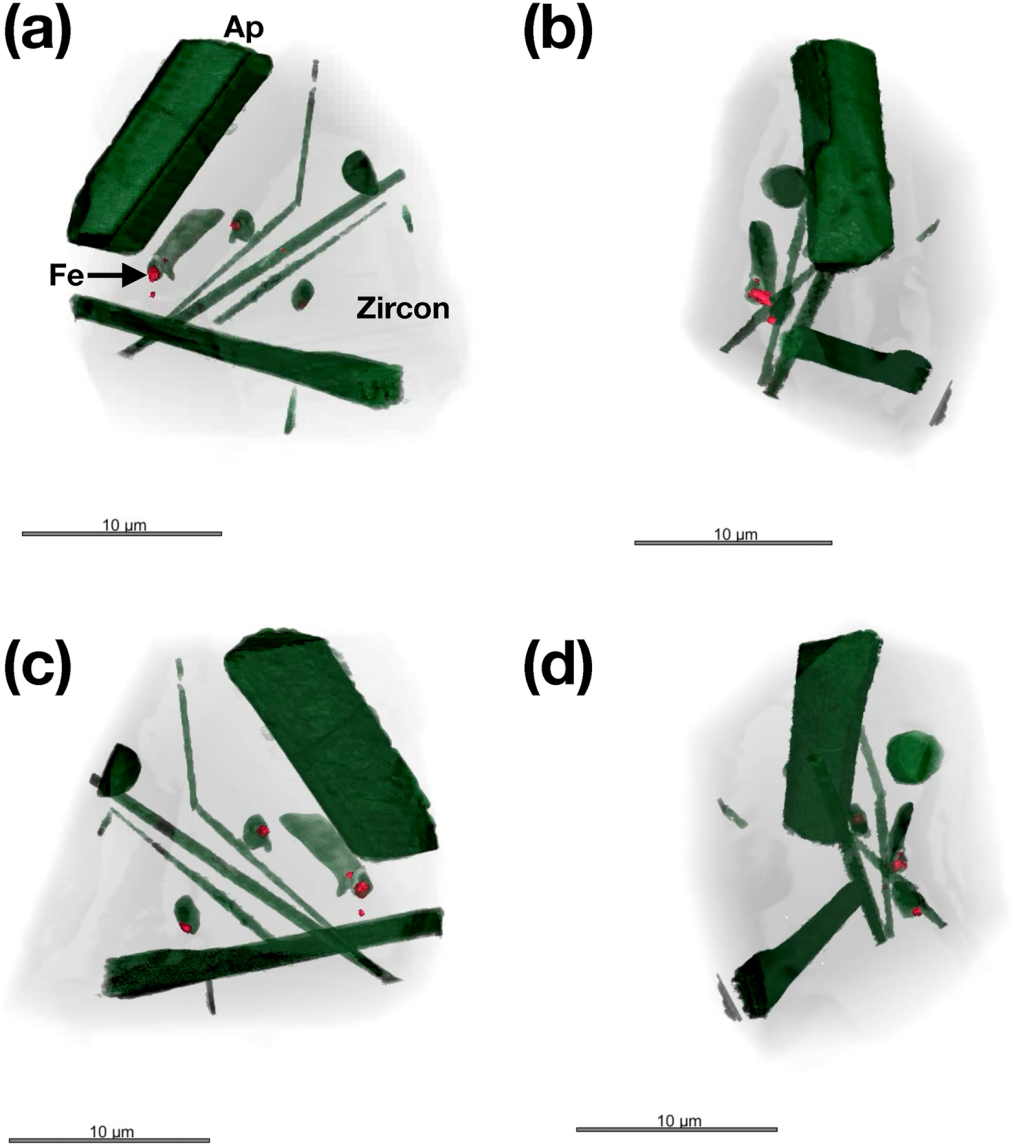
Internal structure of crystal RF1. Apatite (Ap) inclusions are highlighted in green. Fe-rich inclusions are highlighted in red. Images in (**a**–**d**) are related by 90° rotation.

**Figure 5:**
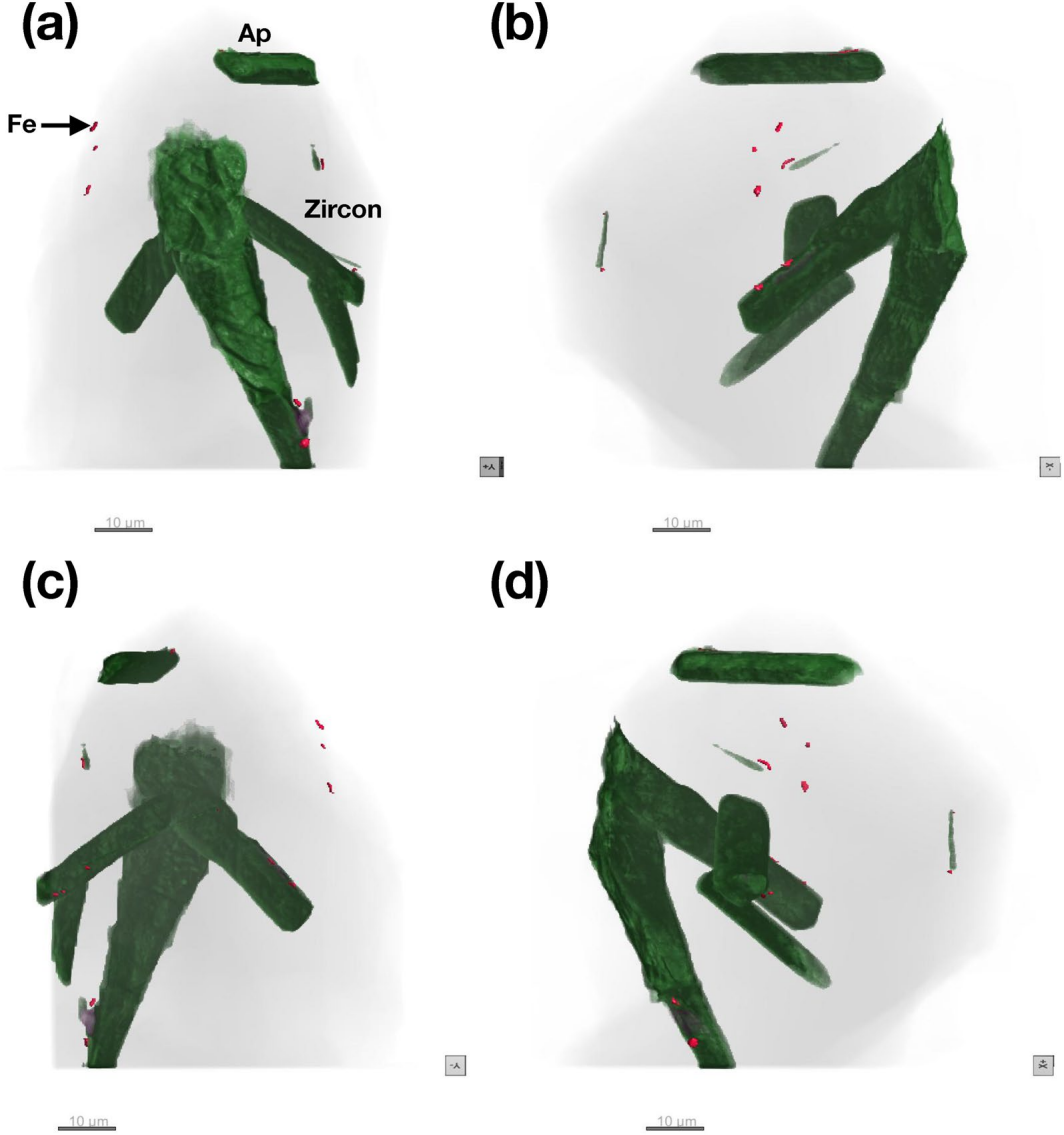
Internal structure of crystal RF2. Apatite (Ap) inclusions are highlighted in green. Fe-rich inclusions are highlighted in red. Images in (**a**–**d**) are related by 90° rotation.

**Figure 6:**
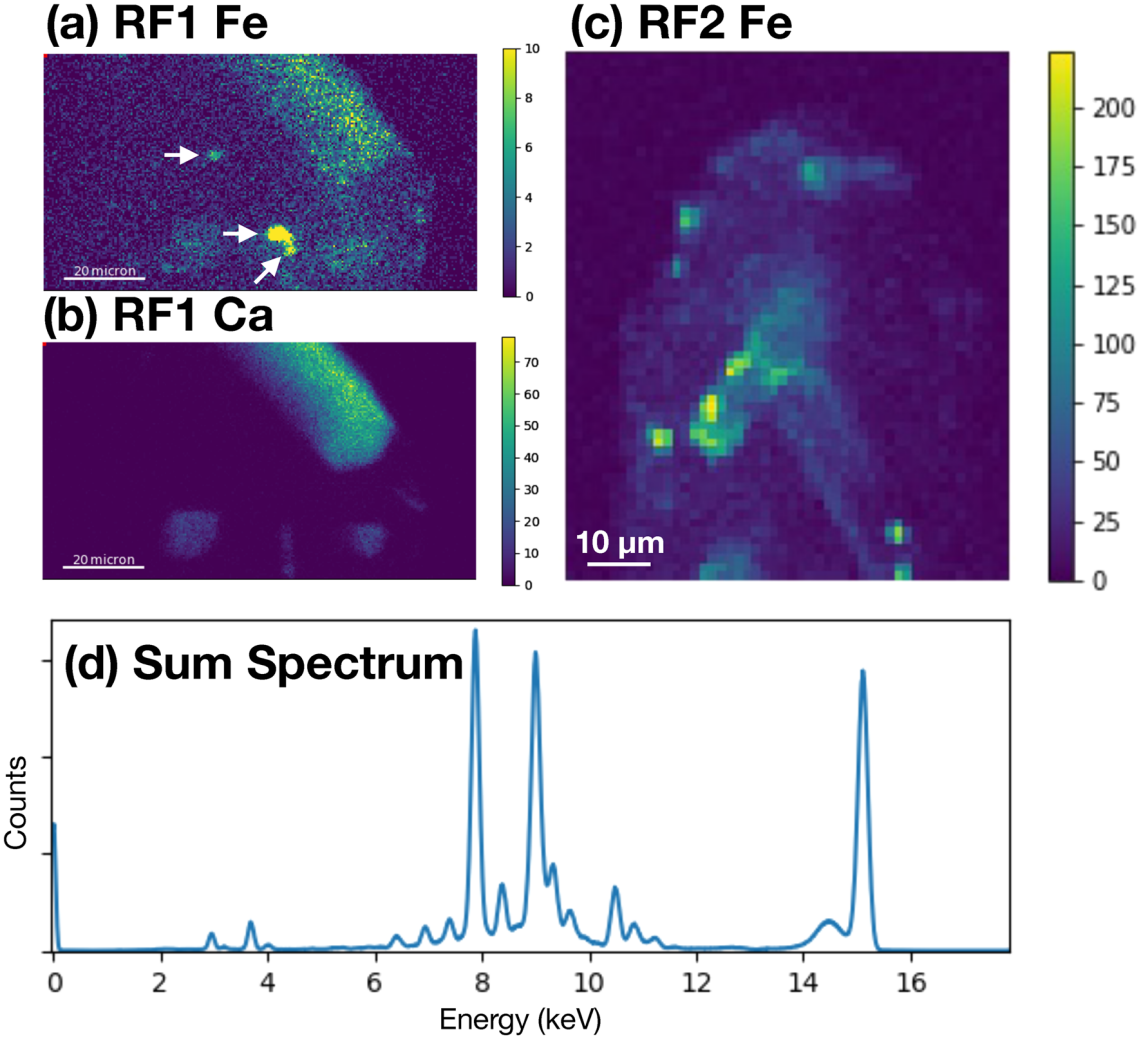
2D chemical mapping of crystals RF1 and RF2 using X-ray fluorescence. (**a**) XRF raster scan of crystal RF1 at the Fe Kα signal (6.4 keV). Intensity scale has been enhanced to highlight weaker features. Note that the XRF detector is placed on the right hand side of the samples, so that features appearing on the left-hand side are attenuated due to self-absorption of emitted X-rays as they travel through the zircon. White arrows, from top to bottom, indicate putative magnetite inclusions appearing in Fig. 9a, b, and c, respectively. (**b**) XRF raster scan of same region in (**a**) using the Ca Kα signal (3.69 keV). Intensity is show at full scale. (**c**) XRF raster scan of crystal RF2 at the Fe Kα signal (6.4 keV). The orientation shown corresponds approximately to that shown in Fig. 5a.

**Figure 7:**
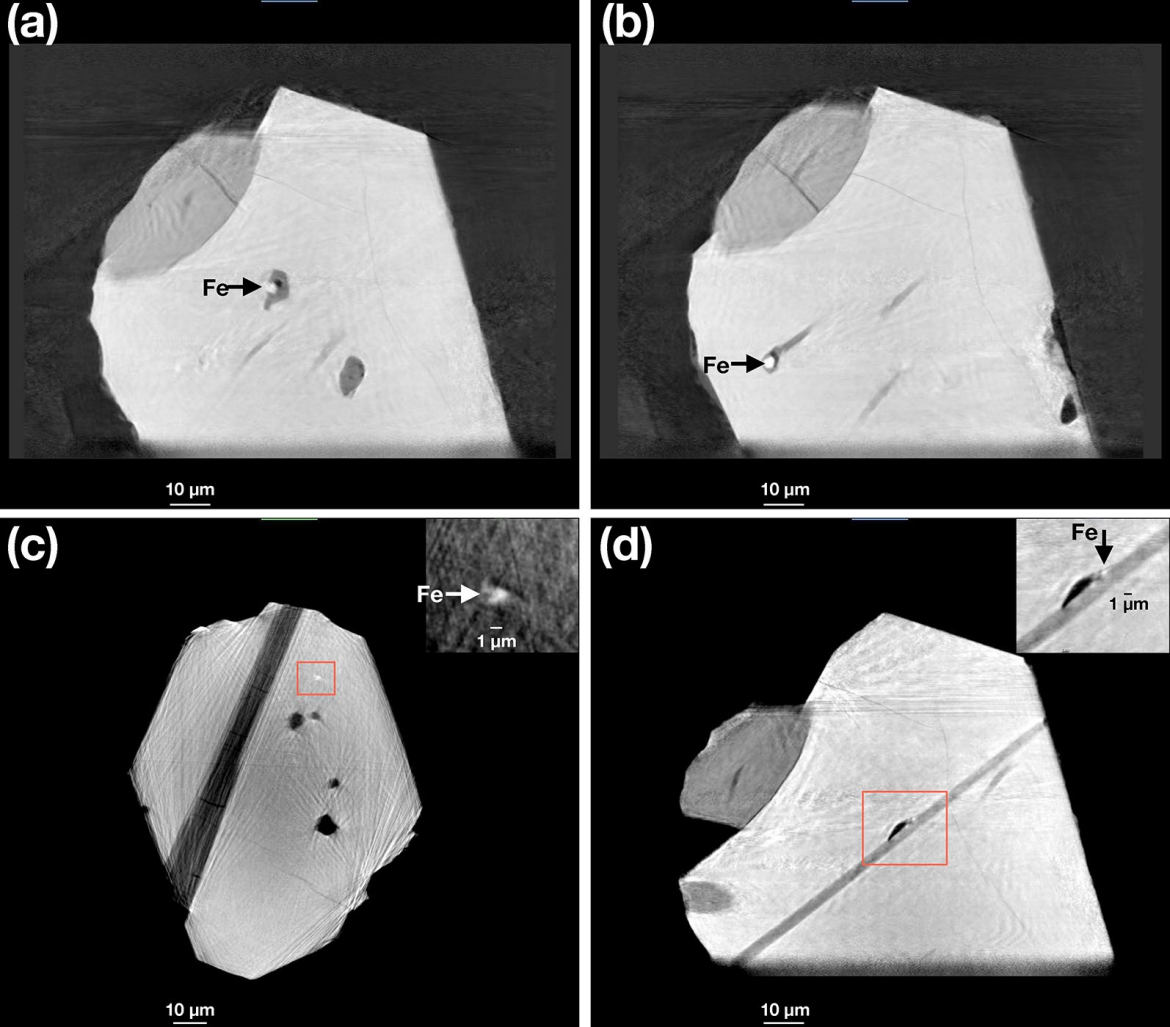
Selected 2D slices through the 85 × 85 × 85 nm voxel phase reconstruction of RF1. Fe-rich inclusions (bright) are indicated by the arrows. The ~ 1 µm-sized particle in (**c**) is not associated with other inclusions or cracks and is therefore confidently identified as a primary inclusion. A magnified and contrast-enhanced image of the region indicated by the red box is inset.

Visualization of the internal microstructure using the 85 nm reconstructions for RF1 and RF2 are shown in Figs. 4 and 5, respectively. Representative XRF projections showing the distribution of Fe and Ca (RF1) and Fe (RF2) are shown in Fig. 6a, b and c, respectively. 2D slices through the 85 nm phase reconstruction of RF1 are shown in Fig. 7, highlighting specific features of interest. Phase images of the zircon host are homogeneous (e.g., Fig. 7b), with no evidence of internal density variations caused by chemical zonation, radiation damage, or recrystallization. Given that such features have been shown to be readily detectable using X-ray nano-tomography (e.g. Figures 1–3 in Suuronen and Sayab^26^), we consider the lack of heterogeneity to be a genuine feature of the crystals rather than a failure of the method to detect it. The only microstructural features observed within the zircon host itself are the occasional fine, sharp cracks, which are not associated with any subsequent alteration or secondary mineralization (e.g., Fig. 7b). Apatite inclusions with a wide range of sizes, aspect ratios and orientations are observed in both crystals. One large apatite inclusion in RF1 lies parallel to a growth surface, whilst the remaining ones are randomly oriented within the interior with no obvious preferred orientation. Cracks and inclusions of a less dense mineral phase are observed in some apatite inclusions, consistent with EDX analysis showing the presence of alkali feldspar (e.g., Fig. 7a). Dark regions with a phase equivalent to that of the crystal exterior are often associated with the apatite-zircon interface, and interpreted here as pore space. The vast majority of Fe-rich inclusions are found to be associated with the interface between apatite, pores and the zircon host (e.g., Fig. 7d and inset), or else appear at the surface of the zircon crystal. The close association of Fe and Ca is particularly evident from the XRF projections presented in Fig. 6. However, at least one Fe-rich inclusion was confidently identified as being embedded entirely in the zircon host, free from intersection with any other microstructural features or inclusions (Fig. 7c and inset).

**Figure 8:**
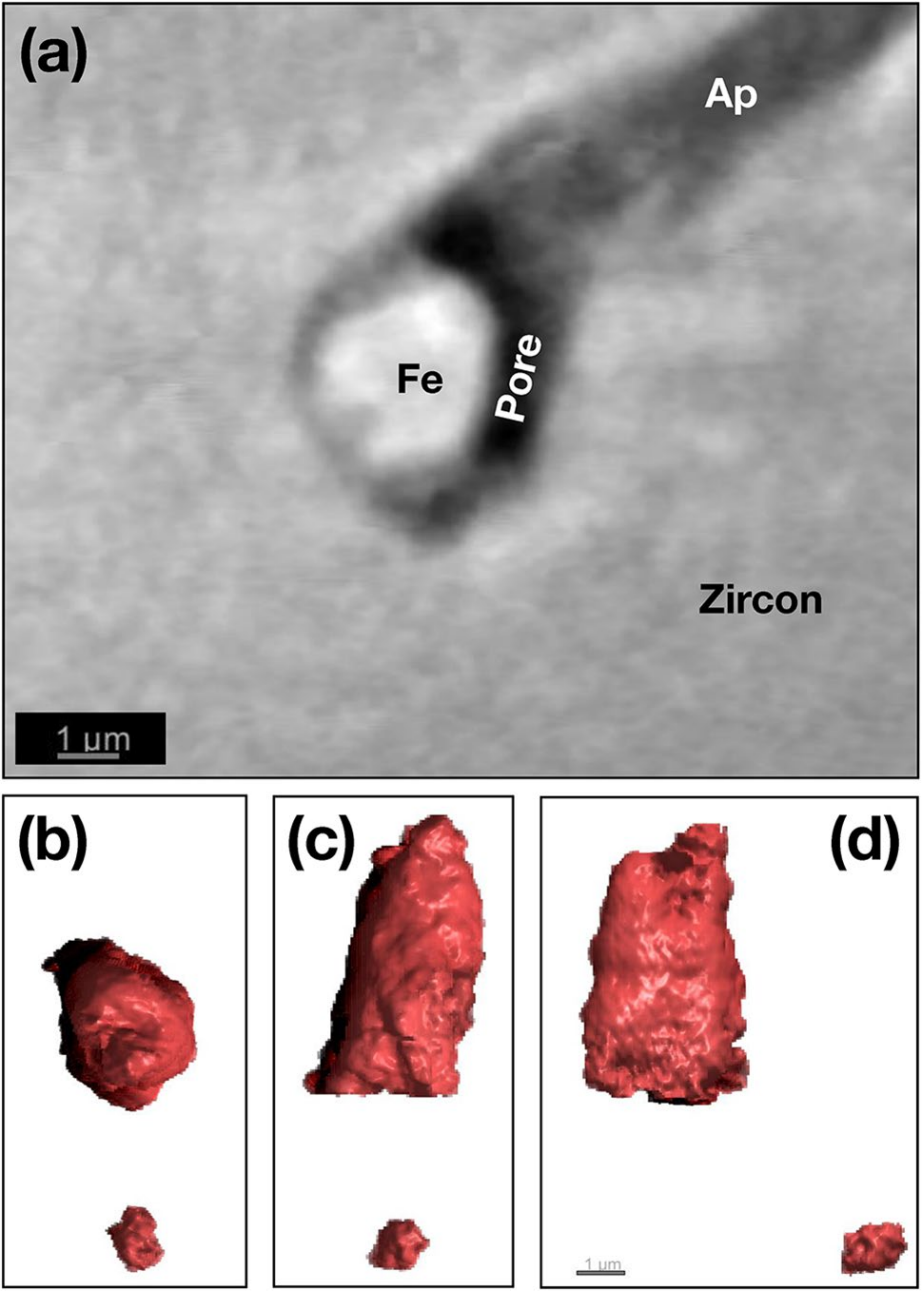
Putative magnetite crystal observed using the 20 × 20× 20 nm voxel phase reconstruction of crystal RF1. (**a**) 2D slice highlighting the putative magnetite crystal shown in Fig. 9b. (**b**–**d**) Orthogonal projections of the 3D reconstruction of the particle shown in (**a**), revealing a bullet-shaped morphology. The small particle below it corresponds to the isolated bright feature shown in Fig. 7c.

The region surrounding the Fe-rich inclusion highlighted in Fig. 7b was investigated in greater detail using the 20 nm voxel reconstruction (Fig. 8). The Fe-rich inclusion (bright) occurs at the termination of an apatite inclusion (dark grey) and is also associated with an adjacent region of pore space (black). The particle has an elongated bullet shape with length ~ 5 µm and maximum width ~ 3 µm, a roughly hexagonal cross section and total volume of 31 µm^3^. Nearby is the particle mentioned above that is entirely embedded in the zircon host, which has an ellipsoidal shape with length 1.6 µm, width 1.1 µm and total volume 0.7 µm^3^.

**Figure 9:**
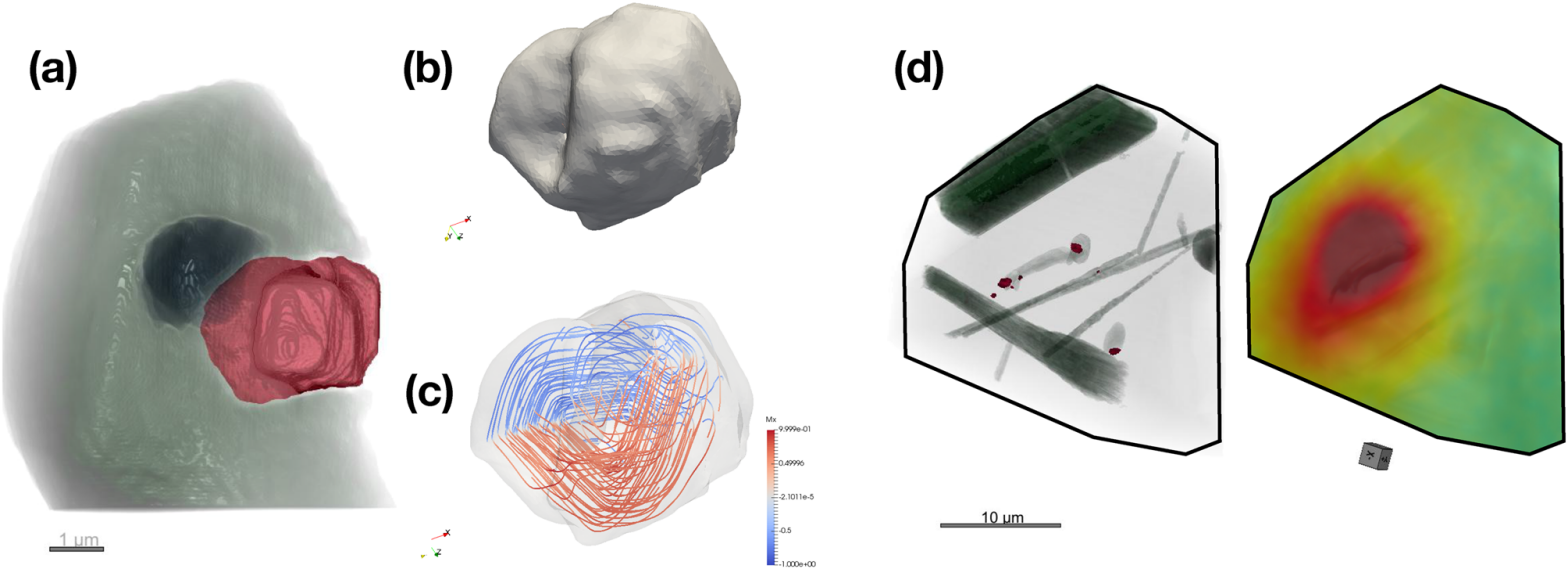
Fe-rich inclusion observed using the 5 × 5 × 5 nm voxel local ptycho-tomographic reconstruction. (**a**) 3D rendering highlighting the Fe-rich inclusion (red) shown in Fig. 7a. Light grey feature is apatite. Dark feature is void space or other low-density material. (**b**) Smoothed, finite-element mesh with average 60 nm mesh size of the magnetite particle in (**a**) used for micromagnetic simulations. (**c**) A typical remanence state obtained by energy minimization from a random seed illustrating the MV-MD domain state. Note that this state should be taken as broadly illustrative only, given that the mesh size used is far greater than the exchange length of magnetite (10 nm). (**d**) Correlation between internal microstructure (left) and QDM magnetic field map for an SIRM state (right) for RF1.

The Fe-rich inclusion highlighted in Fig. 7a was targeted for local tomographic reconstruction using 5 nm voxels (Fig. 9a). The visualization highlights the associated apatite inclusion (grey) and pore space (black). The inclusion is an oblate toroid with width 3 × 3 µm, height 2 µm and an ~ 0.5 µm diameter hole. Assuming that this inclusion is magnetite, the level of detail available in the local tomographic reconstruction is more than sufficient to create a high-quality finite-element mesh that captures the complex morphological features of the particle (Fig. 9b). Due to memory constraints, the resolution of the resulting mesh had to be lowered to 60 nm in order to be run using MERRIL^27^. This is much larger than exchange length of magnetite (10 nm), and so the results presented in Fig. 9c should be taken cautiously as broadly illustrative of the predicted multi-domain (MD) state. The good correlation between the strongest magnetic signals observed using QDM and the positions of these particles (Fig. 9d), indicates that they are capable of carrying a significant saturation moment.

### Resolution limits for detecting Fe-rich inclusions in zircon

To explore the limits of detectability for Fe-rich inclusions (assumed to be magnetite in these image simulations) using X-ray ptychography, and how the size of the reconstructed particles may be affected by the phase-retrieval and reconstruction processing, a synthetic 85 nm dataset containing a mixture of magnetite, apatite and void space embedded in zircon was created and processed in a manner that replicates as closely as possible the treatment of the experimental data (Fig. S3). A 2D slice through the model structure, centered on a 340 × 340 nm particle of magnetite embedded in zircon (4 × 4 modelled pixels), is shown in Fig. S3b. To the left is a larger 1.5 × 1.7 µm particle of magnetite (18 × 20 modelled pixels). To the right is a 1.4 × 1.5 µm slice of magnetite located one 85 nm slice below the plane of the section, and therefore not visible in Fig. S3b. The resulting reconstruction of this layer is shown in Fig. S3c. The larger particle to the left is faithfully reconstructed with dimensions that match the model input. The small central particle is clearly detected and resolved, but significantly broadened compared to the model input, and surrounded by a ring of lower intensity compared to the background. An intensity profile taken along the yellow line in Fig. S3b is shown in Fig. S3c. The full width at half maximum (FWHM) of the reconstructed particle is 751 nm, more than double the size of the model input. A similar analysis of a 170 nm particle of magnetite embedded in apatite produced a reconstructed FWHM of 460 nm. The broadening effect is seen also by the fact that signals from the larger particle on the right, which lies below the level of this slice, are clearly visible.

**Figure 10:**
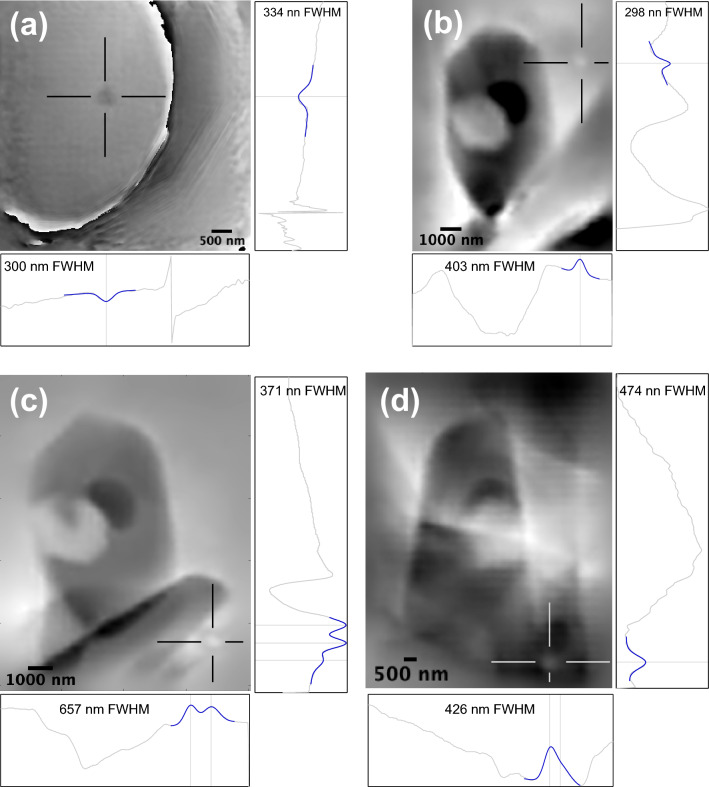
Highlighting the smallest visible Fe-rich particles in 2D ptychographic projections. (**a**–**d**) Wrapped phase projections obtained at 5 nm resolution from crystal RF1. Cross hairs highlight confirmed particles that translate left-to-right systematically with the projection rotation angle. Grey curves show vertical and horizontal profiles, centered on the highlighted particle. Blue curves show the result of least-squares fits to the local phase signal using either one, two or three gaussians and a linear background. Fitted gaussian peak positions are indicated by the vertical or horizontal lines. The FWHM of the peak corresponding to the highlighted particle is indicated. The projections in b, c, and d focus on the region reconstructed in Fig. 9.

The practical limits of particle detectability were explored using the 5 nm ptychographic projections of crystal RF1 (Fig. 10). The use of the raw projection images improves detectability by (a) avoiding additional noise associated with the 3D reconstruction and (b) increasing the signal associated with each particle, which in a projection image derives from the entire projected thickness of the particle rather than a single slice through it. Isolated particles within the limited field of view of the 5 nm projections were identified by looking for features that shifted horizontally systematically with changing projection angle. Four sub-micron particles were identified (Fig. 10). The improved signal-to-noise, compared to Fig. S3d, is clearly seen in the horizontal and vertical phase profiles through the highlighted particles. Particle sizes of ~ 300 nm are clearly resolved (Fig. 10b). Particles of this size are sampled over ~ 60 pixels and so are unlikely to suffer from the broadening error discussed above for particles that are only a few pixels wide. Given the improved signal-to-noise, we consider it a conservative estimate that a 20 × 20 × 20 pixel (100 × 100 × 100 nm) particle of magnetite in zircon, could be resolved using this method with the existing complications (broadening errors). We observed that particles in the range 300–400 nm that were resolved in 5 nm HR mode were not resolved in S mode, even in reconstructions with 21 nm pixel size (Fig. S7). Further analysis showed that along with fundamental parameters determining pixel/voxel size, photon flux plays a major role in determining the resolution limits of ptychographic reconstruction (Fig. S8). To test our hypothesis, we carried out extensive ptychographic simulations employing a 2D synthetic 5 nm phantom (Fig. S5a, b). Photon intensity was varied as a parameter to see its effect. We observe that with the existing geometry, we could have resolved features corresponding to 200 nm spheres (Fig. S6), whereas the intensity of incident photons would need to be scaled up by a factor of 20 to resolve 100 nm particles. We have further carried out simulations with a similar phantom containing features as small as 50 nm. We were (barely) able to resolve the 50 nm feature at 100 scaling (Fig. S11).

## Discussion

### Assessing the paleomagnetic potential of zircon

It is proposed that magnetite inclusions in zircon acquire a paleomagnetic remanence as they cool through their Curie temperature following the crystallization of their zircon host^13^. If a high-temperature component of this primary magnetization can be separated from metamorphic overprints acquired post crystallization, then it may be possible to recover information about the intensity of ancient magnetic field, tied to the age of zircon crystallization obtained using U–Pb geochronometry. The use of zircon single crystals as paleomagnetic targets has aroused a great deal of interest and debate since they were first used to search for evidence of the Hadean geodynamo^13^. Detrital zircons more than 4 billion years old from the Jack Hills, Western Australia provide one of the few tangible records of Earth’s Hadean eon. The survival of such ancient crystals is attributed to the remarkable thermal and chemical stability of zircon, which makes them a good prospect for encapsulating primary inclusions of ferromagnetic minerals such as magnetite and protecting them from subsequent alteration or replacement. Much of the debate surrounding this work revolves around the primary versus secondary nature of the magnetic inclusions – a debate that would benefit greatly from nanotomographic imaging of inclusions and their relationship to other primary and secondary microstructures^6,28–35^.

Although visualization of magnetic particles is not always diagnostic of the timing of magnetization, it can provide strong constraints in some cases and useful context in nearly all cases. For example, recent QDM imaging of ferromagnetic sources in the 3.2 and 3.3 Ga Honeyeater and Kunagunarrina Volcanics of the East Pilbara Terrane^36,37^ has shown association of the NRM-carrying ferromagnetic minerals with a well-defined set of hydrothermal alteration products, thereby (1) showing the secondary origin of the magnetic carriers and (2) providing an age of magnetization interpreted to be in conjunction with radiometric dating of the hydrothermal assemblage. Combined QDM and transmission electron microscopy imaging of two 3.97 Ga zircons from the Jack Hills, Australia^6,32^ identified secondary magnetite produced via a pipe-diffusion mechanism, whereby Fe diffuses into radiation damaged zircon along the cores of dislocations and is precipitated inside nanopores, and during low-temperature recrystallization of radiation-damaged zircon in the presence of an aqueous fluid. The late, secondary addition of Fe into these zircon crystals was subsequently confirmed using Atom Probe Tomography^33,38^ disputes these findings and used a combination of paleomagnetic microconglomerate tests and microstructural analysis to identify primary magnetite located at the intersection of stress-induced cracks that cut across internal zoning and primary magnetite inferred to be inside multiphase inclusions within Jack Hills zircons. In any case, spatial association of ferromagnetic minerals with other mineral phases and microstructural features, in conjunction with high-resolution paleomagnetic tests, can help provide evidence to support a primary versus secondary origin. One way that nanotomographic imaging could further contribute to this debate is the detection of remanence anisotropy at the region-of-interest scale, which ideally should be corrected for when performing high-resolution paleomagnetic tests (e.g.^7^). Recent studies combining nanotomography with micromagnetic modelling have demonstrated how anisotropy can be identified and calculated from first principles^39–41^. Accounting for anisotropy is particularly important in assessing the results of paleomagnetic microconglomerate tests. For example, it has been shown that protracted remanence acquisition by single-crystal zircons in the presence of a reversing field can create remanence directions scattered around a great circle distribution^33^, leading to a false positive microconglomerate test for randomness. If additional remanence scatter due to strong remanence anisotropy were also present, then distinguishing true versus false positive microconglomerate test results might become very difficult. A potential next step in this area would be to perform a combined nanotomography and microconglomerate study on a sample known to carry a secondary remanence to determine whether false positive microconglomerate tests are common, and if their incidence rate can be related to the underlying anisotropy of the magnetic particle ensemble.

Here we observe Fe-rich inclusions primarily associated with the interface between apatite inclusions, pores and the zircon host. The inference that these Fe-rich inclusions are likely magnetite is based on (i) the knowledge that natural remanence in the Bishop Tuff zircons is carried by magnetite or low Ti titanomagnetite based on the observed range of blocking temperatures^25^; (ii) their strong Fe signals seen in XRF maps; (iii) their correlation with strong magnetic signals observed in the QDM maps; and (iv) the good agreement between the observed and simulated bright ptychographic phase contrast, which confirms their refractive index is in the range expected for dense Fe-oxide (rather than, say, Fe-bearing silicate) minerals. The strong association of Fe-rich inclusions with the interface between apatite and zircon could be explained by one of following endmember hypotheses: (i) magnetite and apatite grew separately as primary phases in the magma, magnetite adhered to the surfaces of the apatite, and the magnetite-coated apatite was then encapsulated as primary inclusions within the growing zircon; or (ii) apatite grew in the magma and was encapsulated as primary inclusions within zircon, Fe infiltrated the zircon at some later time, either exploiting the interface between apatite and zircon, or some other microstructural feature, as a fast diffusion pathway to precipitate secondary magnetite. On balance we favor the first hypothesis in this case for the following reasons: (i) several examples of the magnetite/apatite association (e.g. Figure 9a) are found that have no visible connection to the exterior surface of the zircon (e.g., via intersecting cracks) and (ii) there is no evidence of radiation damage, alteration, recrystallisation or deformation in the host zircon or apatite that could provide alternative pathways for Fe infiltration. A caveat is that X-ray tomography cannot detect the presence of individual dislocations, so it is not possible to rule out completely a secondary origin of magnetite growing inside pores fed by pipe diffusion of Fe along dislocation cores, as observed directly by Tang et al.^32^ in Jack Hills zircon. Given the relatively pristine and undeformed nature of these 767.1 ka zircons, however, we consider it less likely that this mechanism would operate on a significant scale in this case.

Limits on detectability for magnetite particles in zircon mean that a significant proportion of the magnetic remanence carriers may be missed using this method in S mode. An absolute lower limit on the missing volume of magnetite can be estimated from the isothermal remanent moment of the zircons. Values of 1.03 × 10^–10^ Am^2^ for RF1 and 7.6 × 10^–11^ Am^2^ for RF2 were obtained here by upward continuing the QDM magnetic field maps (Figs. S1b and e) and then fitting to dipolar field model (Section "Sample selection and initial characterization"). Assuming that all magnetite particles are in a state of complete saturation with a room-temperature saturation magnetization of 480 kA/m, a minimum volume of 158–215 µm^3^ of magnetite would need to be visible in the reconstructions to account for their magnetic moment. This compares with the actual volume observed of ~ 58 µm^3^ for RF1 represented in Fig. 4 and ~ 33 µm^3^ for RF2 represented in Fig. 5. With more realistic assumptions regarding the remanent state of the carriers (e.g., 1–10% of saturation for typical PSD-MD grains^16^), the missing volume could be one-to-two orders of magnitude greater. Clearly, a significant volume of magnetite is missed in the 85 nm ptycho-tomographic reconstructions (S mode). To understand the limitations of S mode, we looked at the features that were resolvable in the HR mode but were not resolved in S mode (Fig. S7). Investigating the far-field diffraction patterns corresponding to regions of interest shown in Fig. S7, one can see that the scattering signal corresponding to feature sizes for a given d spacing is 100 times larger in the case of HR mode. Even at 250 nm d spacing, one has a signal of 100 counts in the case of HR mode while it is barely few counts in S mode (Fig. S8). To validate our findings, we carried out ptychographic reconstructions from modified data sets wherein the data sets were scaled down (multiplied) by a factor. The factor varied between 1.0, 0.9, 0.8, 0.7, 0.5, and 0.2 where 1.0 corresponds to the original dataset. We observe that the feature slowly vanishes with reduction in photons. As the signal corresponding to particle in question becomes smaller and smaller, the feature eventually vanishes in the ptychographic reconstruction (Fig. S9).

While the limitations of S mode on one hand could be seen as a disappointing result, our analysis does confirm (at least indirectly) that the majority of the remanence carriers in these zircons must be significantly smaller than 340 nm and, therefore, are in the SD-SV-MV size range considered to be reliable remanence carriers with high blocking temperatures. Of course, without seeing the particles directly, it is not possible to comment on their relationship to other microstructural features that might have a bearing on the issue of primary versus secondary origin. Ptychographic confirmation of the existence of primary ~ 300 nm Fe-rich inclusions entirely embedded in zircon (Fig. 10) provides direct support for the presence of at least some primary inclusions, and, therefore, that the zircon single-crystal paleomagnetism approach is, in principle, a viable route to acquiring reliable paleointensity data. Zircons from the Bishop Tuff carry natural remanent moments of the order 10^–13^ to 10^–12^ Am^2^, which are sufficient to record information with a 10° uncertainty for directions and a 10% uncertainty for intensity according to the statistical analysis of^1^. One might have resolved smaller inclusions in the range of 200–100 nm by increasing photon flux/frame by a factor of 20. While this has practical implications, which will be discussed in upcoming sections, the use of FIBnt and/or correlative transmission electron microscopy to confirm directly the presence of primary magnetite in the sub 200 nm range might also be introduced as a necessary step in the nanopaleomagnetic workflow.

### Dual-mode ptycho-tomography

We have demonstrated the utility of dual-mode methodology in extracting the optimum amount of information that is practically possible in the context of zircon problem. The effectiveness with which dual-mode ptycho-tomography can solve a given imaging problem will ultimately depend on the ability of each mode be deployed at different ends of field-of-view/resolution spectrum. S mode is effective in surveying the larger picture while HR mode can zoom into regions of interest. Our simulations demonstrate the limits of capabilities in both the modes. While we show that 100 times more flux would resolve features in the single-domain regime with the existing setup, obtaining more flux is not straightforward. Firstly, detector limitations will require a switch from 12-bit acquisition mode (faster acquisition mode, maximum photons: 4906) to 24-bit acquisition mode (slower acquisition mode, max photons: 16,777,216). There are fundamentally two ways of obtaining more photons: increase flux or increase acquisition time. If one takes the former route, photon flux and coherence are usually mutually exclusive, and one will have to balance these two factors effectively to obtain optimal results. The second route to increase acquisition time by a factor of 100 times will make the proposed experiment practically untenable. One can find a middle ground such that we image a smaller field of view with larger acquisition time, but this constrains the amount of complementary information that can be obtained from magnetically active regions of interest. Sample/beam instabilities, additional sources of experimental noise can also be a limiting factor when trying to resolve small (50–100 nm) features. In summary, while our study shows that dual-mode ptycho-tomography is very promising for nano-paleomagnetic applications and beyond, one will have to balance at least 3 parameters (photon flux, coherence length and experimental time) to obtain optimum results.

### Outlook

The direct observation of primary Fe-rich inclusions (assumed to be magnetite) in zircon presented here, and the indirect proof that the majority of magnetite inclusions are in the size range of reliable remanence carriers, provides renewed cause for optimism that the single-crystal zircon paleomagnetic method is viable. Given the paucity of data that exists for the intensity of the geodynamo throughout most of Earth’s history^42^, combined with the sheer number of individual observations that could potentially be made this way, make this a potentially attractive approach^34^. For example, a single sedimentary rock may yield many thousands of individual detrital zircons with a continuous spread of crystallization ages covering millions (perhaps even billions) of years of Earth history. With modest modifications to existing technologies, a single 100 × 100 mount containing 10,000 such crystals could be scanned using a scanning SQUID microscope in a fraction of the time that would be needed to measure an equivalent number of bulk samples, with each crystal providing a unique window on the geodynamo at a specific crystallization time. Such radical approaches may, in the near future, provide the step-change in the quantity and quality of observational data that is needed to study the long-term evolution of the geodynamo. Such data are crucial to resolving debates about the thermochemical history of the Earth, the evolution of the Earth’s core, and role of mantle convection in regulating the properties and behaviour of the Earth’s magnetic field^29,43^. This approach will only work, however, if we have sufficient confidence in the nature and origin of the remanence carriers acquired through a combination of microscopic observations and paleomagnetic tests. We have shown that ptycho-tomography has a significant role to play in the workflow that is required to provide that confidence. Its role is likely to get progressively more important with increasing age of the zircons, as the microstructures associated with radiation damage and recovery get more complex, and the formation of secondary magnetite gets ever more likely. However, given the practical detection limits, we strongly recommend the use of focused ion beam nanotomography and/or correlative transmission electron microscopy to confirm directly the presence of primary remanence carriers in the sub 300 nm range as part of targeted paleomagnetic workflows (e.g.,^6,32,39,40,44,45^).

Beyond zircon, we envisage X-ray ptycho-tomography could play a major role in the characterization of other important single-crystal targets, including quartz, feldspar, olivine, pyroxene, rutile and baddeleyite in terrestrial and extraterrestrial rocks, chondrules, calcium-aluminium-rich inclusions^46^ and aqueously altered matrix regions in chondritic meteorites. The spatial resolution and detectability of magnetite makes ptycho-tomography a suitable approach for the *in-situ* study of giant magnetofossils in marine sediments^47^ and could potentially even detect conventional magnetofossils when combined with the dual-mode approaches outlined here and the increased phase contrast between magnetite and low-density sedimentary grains [e.g.,^29^]. The non-destructive nature of X-ray tomography applied to meteorites is discussed in detail by Hanna and Ketcham^48^, who conclude that no resolvable changes in magnetic moment are observed as a result of typical X-ray tomography measurements and that heating (estimated to be < 1 °C over a 3 h measurement) and mineralogical or chemical alteration are negligible^49^, although some impact on the thermoluminescence properties of meteorites can be expected due to the irradiation^50^. Several studies have shown that under typical conditions for X-ray tomography, there is little to no damage or modification of organic compounds in carbonaceous chondrites^51–53^ and therefore that these methods may be an especially important tool for the characterization of unique samples returned from asteroids and Mars^54^ as well as have major application in the search for the evidence of early life on Earth.

## Materials and Methods

### Sample selection and initial characterization

We sampled a ~ 2 kg block of Bishop Tuff welded ignimbrite from unit Ig1Eb according to the stratigraphic definitions of Wilson and Hildreth^55^. Oxygen isotopic data from this unit suggests a lack of post-emplacement hydrothermal alteration and probable preservation of igneous Fe-oxides^56^. After breaking the block into smaller volumes using non-magnetic BeCu tools, we conducted further crushing using alumina grinding vessels in a Spex Shatterbox^®^ at the M.I.T. Radiogenic Isotope Laboratory. Subsequent manual picking yielded approximately 40 zircons. We previously analyzed these zircons for their paleomagnetic record using the SQUID Microscope, finding paleointensities with < 15% uncertainties that are concordant with bulk sample results^24,25^. Two zircons (RF1 and RF2) that were not part of the paleointensity heating experiments were selected for ptycho-tomography analysis (Figs. S1a, d). These crystals were mounted in a block of non-magnetic EPO-TEK 301 epoxy and given a 0.4 T isothermal remanent magnetization in the out-of-plane direction. We imaged these zircons using a QDM at Harvard University, which took data in vector magnetic microscopy (VMM) mode using a 0.9 mT bias field that was reversed during measurement to reduce its contribution to the field map to < 1 µT^57^. The effective spatial resolution of the QDM maps (Figs. S1b, e) was 2.4 µm. The isothermal remanent magnetic moment of each crystal was obtained using an upward continuation/dipole fitting approach, yielding 1.0 × 10^–10^ and 7.6 × 10^–11^ Am^2^ for RF1 and RF2, respectively. Dipolarity parameters of 0.81 and 0.90 associated with these fits, respectively, correspond to uncertainties of approximately 10% and 5% in moment^4^. A fit focusing on just the strong magnetic signal observed in the upper left of RF1 (Figs. S1b) yielded 8.0 × 10^–11^ Am^2^ with an estimated 20% uncertainty based on the same method.

Following the QDM mapping, the two crystals were carefully extracted from the epoxy and mounted on sharp tungsten carbide tips using ultraviolet (UV) setting glue (Figs. S1c, f). Secondary electron (SE) imaging (Figs. S2a, c) and energy-dispersive X-ray (EDX) spectroscopy (Figs. S2b, d) of part of the surface of each zircon crystal was performed using a Quanta-650F scanning electron microscope (SEM) at the Department of Earth Sciences, University of Cambridge. Imaging was performed in low-vacuum mode to prevent charging of the uncoated samples at an accelerating voltage of 20 kV (RF1) and 30 kV (RF2). Chemical maps were calculated using the open source programme HyperSpy (10.5281/zenodo.592838) by integrating X-ray counts for the Zr L_α_, Si K_α_, Ca K_α_ and Fe K_α_ peaks. Integration was performed without background subtraction over an integration window equal to twice the full-width at half maximum of the peak. Chemical maps for Fe, Ca and Zr were normalised to their maximum counts and used as the red, green and blue channels, respectively, of the RGB images shown in Figs. S2b, d.

### X-ray ptycho-tomography and X-ray fluorescence measurements

The term X-ray nano-tomography covers a range of X-ray 3D imaging techniques, including X-ray holotomography^26^ and X-ray ptycho-tomography^17,58^, that use coherent beams of X-rays generated by a synchrotron to create high-quality phase-contrast images of the sample, in addition to the absorption-contrast images normally associated with standard X-ray tomography methods. Spatial resolution of 45 nm with voxel size of 17 nm over a 30 × 16 µm field-of-view was obtained for a porous sandstone sample using X-ray ptycho-tomography by De Boever et al.^58^. The current record for 3D spatial resolution is 16 nm^59^. While radiation damage is always a possibility, this is usually associated with soft matter (polymers, biological cells, soft tissue etc.); minerals/metals are far less susceptible to radiation damage. Similarly, although many X-ray beam lines use magnets for convenience as part of the sample mounting assembly, their use is not essential, and it is possible, in principle, to perform these measurements without exposing the samples to a magnetic field prior to paleomagnetic analysis.

Unlike its sister techniques, such as transmission X-ray microscopy (TXM)^60,61^, Fourier transform holography (FTH)^62^ or scanning transmission X-ray microscopy (STXM)^63^, the resolution from the ptychographic imaging modality doesn't depend on the focusing optics or the reference aperture, but rather on the detectable signal contained in the measurable inverse space. Resolutions as high as 5 nm and 17 nm were previously reported in the soft (energy < 2 keV) and hard (6.5 keV < energy) X-ray regimes, respectively^64,65^. Another major advantage of ptychography is that the object is de-coupled from the illumination function incident on the sample. This means that ptychographic reconstructions are largely unaffected by lens distortions, thus obtaining better quality images compared to other techniques. Ptychography also has the capability to counter partial coherence effects arising from probe/sample instabilities or the inherent partial coherence of the illumination function^66^. Ptychography, however, is a computationally intensive technique, and relies heavily on massively parallelized algorithms to obtain optimum results in practical time scales. Phase-retrieval algorithms, such as difference map (DM) algorithm^21,67,68^ or the ptychographic iterative engine (PIE)^69^, are normally used to obtain the reconstructions.

Although the resolution of ptychography is not strictly dependent on the size of the illumination function, the over-sampling criterion places limits on the maximum size of the illumination function, and thereby on the field-of-view (FOV), which, in turn, limits the pixel size. As a result, one can expect to obtain either high resolution (~ 5 nm pixel size) for small FOV (10–15 µm) or modest resolution (~ 85 nm pixel size) for large FOV (80–100 µm), assuming the same detector is used. Although a combination of high resolution and large FOV could be obtained by employing large detectors at large sample-detector distances, this setup would substantially increase computation time due to the high number of large arrays required by the phase retrieval algorithms. As ptychography is a scanning technique, the FOV and computation time can be reduced by simply choosing a subset of the original diffraction pattern dataset. Tomographic factors also influence the ultimate resolution of the 3D transmission function. To obtain optimal 3D resolution the number of projections required is approximately 1.6 (i.e., π/2) times the number of pixels occupied by the sample within the FOV^70^. A large FOV at 5 nm pixel size necessarily contains a large number of pixels, requiring a large number of angular projections to obtain optimal 3D resolution. These experimental, computational, and practical considerations make high-resolution, large FOV ptycho-tomography challenging. To mitigate the above factors and obtain a full picture without compromising FOV or resolution, we develop here two separate but overlapping modes: a survey mode (S mode) that yields large FOV with low to moderate resolution, depending on detector (crop) size, and a high-resolution mode (HR mode) that yields high resolution over a targeted FOV. By employing two separate detectors, one can seamlessly convert from one mode to the other. The survey mode can be used to image large volumes and identify regions of interest which can later be probed in high-resolution mode to obtain maximum information. Survey mode can also be used to probe the broader scale microstructural features of the sample, such as the presence of cracks and other large inclusions. We call this methodology ‘dual-mode ptycho-tomography’, combining two maximalist imaging modalities to extract as much information as practically possible.

All experiments were performed here using the I13-1 coherence branchline of the I13 long beamline for imaging and coherence at the Diamond Synchrotron Source, Didcot, United Kingdom. I13-1 is ideally suited to conduct multi-scale and multi-modal ptychography and ptycho-tomography^71–77^. We employed a hybrid, dual-mode experimental geometry with an X-ray energy of 15 keV. A focusing optics ensemble, comprising a 400 µm diameter blazed Fresnel zone plate, a 60 µm diameter central beam stop and a 25 µm diameter order-sorting aperture was employed to obtain the optimum X-ray illumination function^78^. The Merlin detector was positioned 1.29 m from the sample to record the far-field diffraction patterns in high-resolution mode (HR mode). This geometry allowed us to obtain 5 nm pixel size with 384 × 384 detector cropping. The Excalibur detector was positioned at 14.6 m to conduct ptycho-tomography in the large FOV survey mode (S mode). The larger area of the Excalibur detector enabled imaging at multiple resolutions depending on the choice of detector cropping. Pixel sizes of 85 nm and 21 nm were achieved with 256 × 256 and 1024 × 1024 detector cropping, respectively. We employed optimum overlap parameters and imaged the entirety of each crystal in each case. To satisfy the over-sampling criterion we employed a probe of ~ 1 µm extent in HR mode and ~ 10 µm extent in S mode. In each case we acquired the optimum number of ptychographic angular projections. In S mode we calculated this value depending on the moderate resolution corresponding to 256 × 256 cropping. The Merlin detector was positioned on top of a long-range linear stage, and could be moved in and out of beam, thus giving us the ability to shift between S- and HR modes seamlessly. We employed a Vortex X-ray Fluorescence (XRF) detector positioned perpendicular to the beam propagation to acquire chemical information.

### X-ray ptychography processing and reconstruction

Ptychographic reconstructions were carried out using ‘Ptycho’, a ptychographic reconstruction code based on the Difference Map algorithm coded in the Python programming language^21,67^. The code was parallelised using mpi4py^79^ and can be accelerated by employing multiple computer cores for a given single ptychographic projection. As a single ptycho-tomography dataset may contain 600–1000 ptychographic projections, reconstruction is inherently computationally intensive. A sample ptychographic projection was put through multiple rounds of the algorithmic image reconstruction process to optimise the reconstruction parameters (e.g., number of iterations, Fourier relaxation factor, and X-ray illumination modes)^78^. The optimized parameters were then used for all the ptychographic projections of a given ptycho-tomography dataset. As this process becomes near impractical with conventional computational resources, we employed the ARCHER supercomputing cluster to bring down the processing times from months/weeks to days/hours. Even here, we faced challenges in reconstructing the S mode datasets with 1024 × 1024 cropping owing to the large RAM requirement. In this regard, we were successful in exploiting the information from QDM mapping to identify sub-regions of interest within the larger field-of-view. Only the most important regions were reconstructed at the highest obtainable resolution (21 nm) in S mode. This step reduced the computational challenge and made the reconstructions feasible in practical time frames.

Owing to the ultra-large field-of-view, combined with the dense nature of samples, the ptychographic reconstruction process was particularly challenging in this study. We developed two constraint mechanisms to mitigate these issues. First, the high density of the sample can be mitigated by using higher beam energy (15 keV in this case). Imaging samples at high energies (> 12 keV) opens up exciting possibilities, such as the ability to image thick, dense 3D samples. However, at higher energies there is a possibility that the amplitude and phase-shift maps diverge in their appearance (Supplementary Text). This divergence introduces artifacts primarily in the amplitude component of the object’s transmission function during the ptychographic reconstruction process, ultimately leading to sub-optimal ptychographic reconstructions that are prone to artifacts and a sub-optimal reconstruction of the illumination function. We introduced a Gaussian blurring step that blurs the amplitude of the object’s transmission function before every iteration step. This new constraint improved the ptychographic reconstructions, whereby artifacts corrupting the reconstructions were removed (Supplementary Text). Second, while the aforementioned constraint improved ptychographic reconstructions, the reconstructed illumination function still appeared sub-optimal in some of the reconstructions. The reconstructed illumination function acts as a counterweight in assuring the quality of a reconstruction^78^ and one needs to diagnose/mitigate the factors resulting in sub-optimal illumination functions. We developed a diagnosis technique based on Principal Component Analysis (PCA) that is able to analyze and correct for the root causes of sub-optimal probe function (Supplementary Text). In ptychography the far-field diffraction pattern is formed by interaction between object’s transmission function and a partially coherent probe function. A partially coherent probe can be thought of as the resultant of *N* mutually incoherent, coherent illumination function modes with different amounts of power associated with each mode^66,78^. Usually, an initial illumination guess is made, whereby the primary probe mode (depending on experimental geometry) possesses the majority of probe power, while the remaining modes possess random noise with nominal power. Such an initial guess has too many degrees of freedom to absorb unwanted structure into the illumination function. Over multiple iterations, this aspect corrupts the probe ultimately corrupting the object’s transmission function. We have therefore devised a new constraining methodology in which one starts with a guess where all modes are identical containing identical amount of power. This prevents the probe from getting corrupted, thus improving the ptychographic reconstructions (Supplementary Text).

Tomographic reconstructions were performed using the phase component of the ptychographic projections, which has far greater contrast than the absorption component in the hard X-ray regime. Prior to reconstruction, the phase components underwent multiple pre-reconstruction steps. The datasets were corrected for phase ramp using a combination of automated and manual phase-ramp correction techniques^78^. The projections were aligned in the axial direction, employing automated and manual edge-detection methods^78^. The projections were corrected in the radial direction employing a center-of-mass alignment method^78^. Finally, 3D reconstructions were obtained by employing a modified ‘filtered back projection’ method on the derivatives of the aligned ptychographic phase projections, which circumvents the need to unwrap the phase images prior to reconstruction^80,81^.

### Phantom calculation

To explore the limits of detectability for magnetite particles using X-ray ptychography, and how the size of the reconstructed particles may be affected by the phase-retrieval and reconstruction processing, a ‘phantom’ dataset containing a mixture of magnetite, apatite and void space embedded in zircon was created and processed in a manner that replicates as closely as possible the treatment of the experimental data. The phantom for simulations was a modified 3D Shepp-Logan phantom^70^ containing 1024 × 1024 × 1024 pixels with 85 nm pixel size (similar to the S mode data with 256 × 256 detector cropping). The total extent of the phantom is 87 µm, similar to the sample dimensions imaged in this study. The original phantom was modified by adding ellipsoids of various sizes with random orientations, embedded within, or lying on the edges of, the existing primary ellipsoids. Each ellipsoid is assigned a “refractive index” value based on the chemical phase these ellipsoids are expected to represent.

Ptychography datasets at a given rotation angle (φ) were generated by finding the 2D complex transmission function from the rotated 3D refractive index phantom^78^. The 2D transmission function was multiplied by a complex illumination function (borrowed from one of the experimental datasets) with optimum overlap. As each scan point, the intensity of far-field diffraction (assuming a sample to detector distance of 14.6 m) pattern was saved (256 × 256 cropping) along with the position information of the scan point. This process was then repeated for 1600 equally spaced angles in the − 90° to 90° angular range. It should be noted that the generated datasets were quite ideal in theory and something similar will be very hard to achieve in a real experiment.

The ptychographic phase projection at a given angle was reconstructed using the same algorithmic framework as the experimental datasets. Same process of optimization was employed and all datasets were reconstructed on the ARCHER supercomputer. We employed the same constraint mechanisms in reconstructing simulated datasets and also tested the efficacy of our newly developed constrained mechanisms in terms of quality/resolution of the obtained reconstructions.

The reconstructed phase projections were taken through tomographic reconstruction routine same as the experimental datasets. Automated phase ramp correction was employed, a manual edge detection was used to correct for drifts/shifts in the axial direction and correction in radial direction was done using center of mass alignment method. 3D reconstructions were obtained by employing a modified “filtered back projection” method on the derivatives of the aligned ptychographic phase projections same as the experimental datasets.

### Simulation studies on factors effecting ptychographic resolution

In order to fully understand the limits of resolution especially in the HR mode and evolve a criterion for optimum flux required to resolve features we have carried out extensive simulations. The first step involved engineering illumination functions to accurately depict the experimental conditions. We have achieved this by borrowing the probe functions from the ptychographic reconstruction showed in Fig. 10 c. The amplitude of the borrowed probe was scaled by a given factor Pf_sc_ such that one gets increasing or decreasing photon values for various Pf_sc_. These engineered probes were then used in conjunction with the reconstructed object functions to generate the forward modeled ptychographic data sets. These data sets were then reconstructed using the same algorithmic parameters as the reconstruction shown in Fig. 10 c. The reconstructed objects were then compared to obtain parity between the forward model involving engineered illumination functions and the synthetically scaled experimental data sets (Fig. S10). Once parity was established, we used the same engineered illumination functions to generate ptychographic data sets with resolution phantoms. The resolution phantom contains a series of (6) magnetite spheres embedded midway in a 100 um thick zircon matrix. The diameters are 5000, 1000, 500, 300, 200 and 100 nm arranged in a spiral fashion (Fig. S5). The scaling of the illumination functions varied between 0.1 to 100 in order to fully gauge the effects of photons on the obtainable resolution. In order to further push the understanding on resolution limits we have carried similar studies on a finer phantom with 7 magnetite spheres 5000, 1000, 500, 300, 200, 100 and 50 nm. The scaling of the illumination function was limited to 100 (Fig. S11).

### Segmentation and visualization

Tomographic reconstructions were analysed using the software Dragonfly Pro by ORS Inc. A non-local means (NLM) and/or median filter was applied to the data to reduce noise prior to segmentation. Segmentation was performed using intensity thresholding to highlight individual phases and microstructural features of interest, followed by manual clean up. Smoothing of the segmented surfaces was applied to individual objects to remove outlying voxels and improve the quality of the visualizations, taking care not to visibly compromise their morphological features. 9Visualizations presented throughout the paper show the zircon host (grey), apatite (green), Fe-rich inclusions (red) and pores (black). All features discussed in detail are considered likely to be magnetite due to their association with Fe signals observed in XRF scans and magnetic signals observed in the QDM. However, in the absence of direct diffraction evidence it is not possible to be definitive regarding this phase identification, so we refer to all particles as Fe-rich inclusions throughout.

### Supplementary Information

Supplementary Information.

## Data Availability

The data contained within this paper is freely available as a series of tiff images representing each slice of the reconstructed 5, 21 and 85 nm volumes at https://data.gov.uk/dataset/4734efe3-5633-4b95-9a39-17645c1d51ee/ptychotomography-of-bishop-tuff-zircons-nerc-grant-ne-p002498-1.
