# Supplementary material for: Nanoscale imaging of Fe-rich inclusions in single-crystal zircon using X-ray ptycho-tomography
Source: Sci Rep. 2024 Mar 1;14:5139. doi: 10.1038/s41598-024-55846-4 (PMC10907758; doi:10.1038/s41598-024-55846-4)
Supplement: Supplementary file 1 — Supplementary Information. [file 41598_2024_55846_MOESM1_ESM.docx]

Supplementary Materials for

**Nanoscale imaging of Fe-rich inclusions in single-crystal zircon using X-ray ptycho-tomography**

Venkata S. C. Kuppili, Matthew Ball, Darren Batey, Kathryn Dodds, Silvia Cipiccia, Kaz Wanelik, Roger Fu, Christoph Rau, Richard J Harrison^*^

*Corresponding author. Email: rjh40@cam.ac.uk

**This file includes:**

Supplementary Text

Figs. S1 to S22

Supplementary Text

A negative consequence of ptychographic imaging large, dense three-dimensional samples, such as zircon, at high energies (> 12 keV) is that the object’s phase shift undergoes multiple phase wraps while the amplitude takes the usual Lambert-Beer behavior of exponential decay (Fig S12). As the divergence between amplitude and phase-shift increases, artifacts relating to the crosstalk between amplitude and phase shift appear in the reconstruction (Fig. S13). The artifacts amplify over the course of reconstruction process, ultimately leading to sub-optimal ptychographic reconstructions. The reconstruction process initially reconstructs the “edge” features within the transmission function and the phase-shift does not depict the reality accurately (Fig. S13). Over the course of the reconstruction process, the algorithm (rightly) starts reconstructing the phase wrapping aspect of the phase-shift of the object’s transmission function. The reconstruction process however introduces artifacts into the amplitude of the object’s transmission function (Fig. S14). One can see that while the algorithm rightly reconstructs the phase wrapping of the phase-shift of the object’s transmission function it still introduces pock-marked artifacts into the ptychographic reconstruction. The reconstructed probe also looks sub-optimal (Fig. S15).


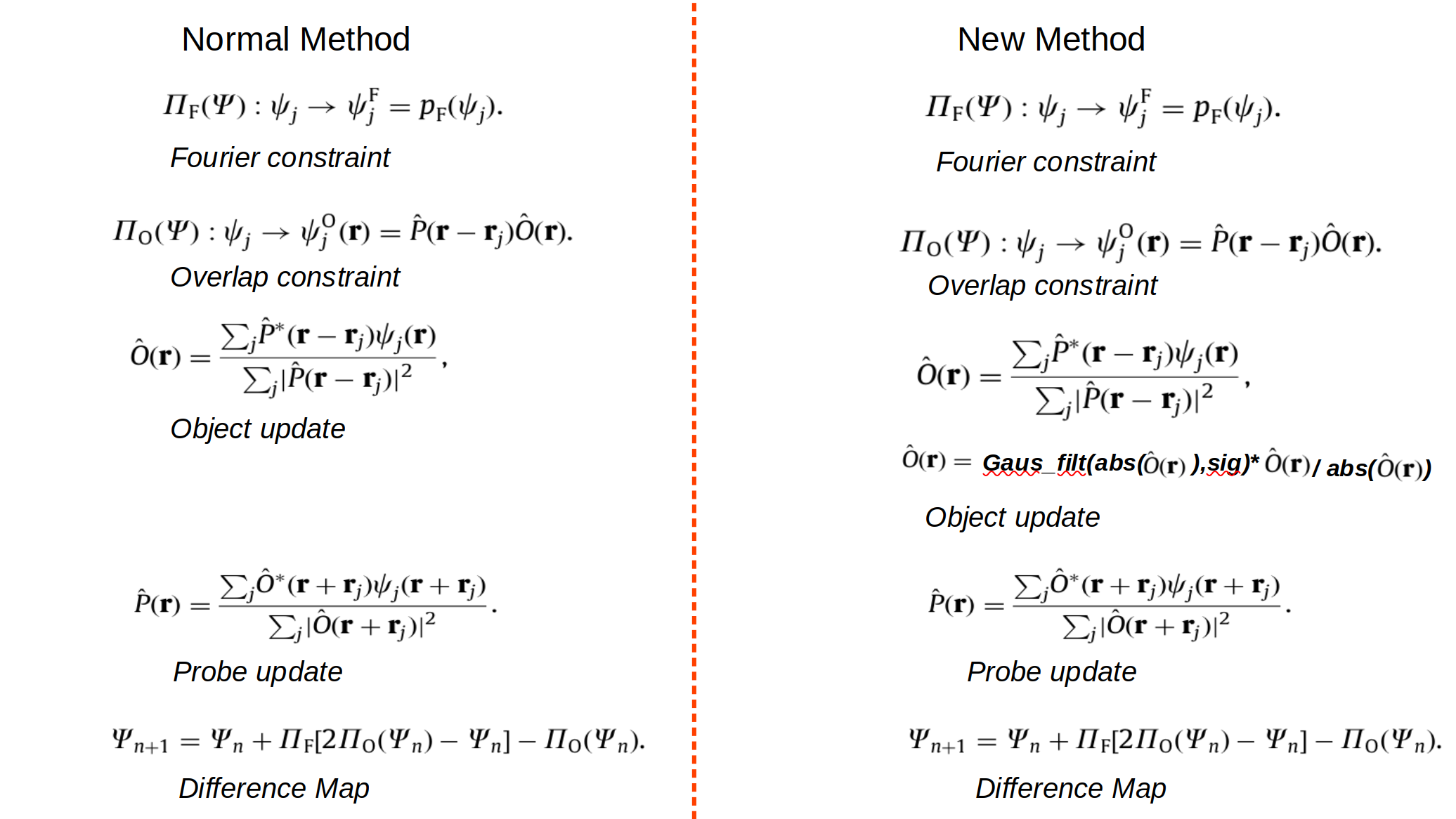
To solve this problem, we introduced a Gaussian blurring operation of the amplitude of the object’s transmission function during the overlap projection (probe/object update) [Thibault et al., 2009, Kuppili 2020]. A Gaussian filter implemented in scipy.ndimage.filters [9] was employed, a standard deviation of 50 pixels was determined as optimum through empirical testing. A comparison of the new method compared to the conventional ptychographic reconstruction method (Thibault et al., 2009) is given below:

Where *P*(*r*) is the complex valued probe function, *O*(*r*) is the complex valued object function, *ψ_j_*(*r*) denotes the *j*^th^ exit wave, where *j* denotes *j*^th^ scan point of the dataset. *Ψ_n_* is the state vector representing (*ψ*_1_(*r*), *ψ*_2_(*r*), *ψ*_3_(*r*),…), here *n* denotes the iteration value. *Gaus_filt* is the Gaussian filter function and *sig* is the standard deviation. *abs* denotes the absolute value function, * is the conjugate operation.

Confirming our assumption that phase-shift contains crucial information, the addition of a Gaussian blurring operation solves most of the problems involving artifacts (Fig. S16). However, the reconstructed illumination function remains suboptimal. An optimal ptychographic reconstruction should accurately represent both the object’s transmission function along with incident X-ray illumination function. The incident illumination function is a time, position invariant entity. All effects that are time, position invariant or whose variation is fast compared to the acquisition of single far-field diffraction pattern can be brought together under the ambit of a partially coherent (X-ray) illumination function [1]. Owing to the reciprocity principle [2,3], effects arising out of sample dynamics [4,5,6], mechanical instabilities and imperfect detectors [7] can also manifest as part of a position/time invariant illumination function. Principal Component Analysis (PCA) can be used as a tool to reduce a complex dataset into a set of mutually un-correlated components [8]. The components themselves encompass the most common features of the entire dataset and have been used in diverse fields, such as face recognition. Taking the example of face recognition, a PCA analysis of a facial recognition dataset will yield a set of eigen faces and eigen values. The primary “eigen face” will contain the most common features that are contained in the entirety of the dataset. Similarly, the illumination function reconstructed by ptychography, assuming that the probe is a position/time invariant complex function, can be estimated by calculating the eigen face of the far-field diffraction patterns acquired during ptychography (Fig. S17). In this way, valuable information regarding the illumination function can be obtained from the dataset even before carrying out ptychographic reconstruction.

This principal can be used to diagnose issues with sub-optimal ptychographic reconstructions, by providing a better initial guess of the illumination function. The conventional method of ptychographic reconstruction uses an initial probe guess where the primary mode (depending on experimental geometry) contains the illumination function possessing the majority of probe power, while the rest is made to possess random noise with nominal power. Such an initial guess has too many degrees of freedom to absorb unwanted structure into the illumination function. Over multiple iterations, this aspect corrupts the probe, ultimately corrupting the object’s transmission function. We have devised a new methodology in which one starts with a guess where all modes are identical containing identical power. The initial guess for the probe can either be simulated assuming a zone plate is being employed or it can be “borrowed” from another reconstruction. In Fig. S19 we illustrate our method using a simulated probe but in our actual reconstructions we borrowed a probe form a different reconstruction. We observed sub-optimal probes only in some projections (in an angular range) probably because of very strong edge scattering etc. Therefore, we were able to borrow a probe from a good reconstruction and use it as an initial guess, but with identical mode structure. We used the same strategy for all the angular projections, yielding good reconstruction results for all of them. Our approach prevents the probe from getting corrupted, thus improving the ptychographic reconstructions.

The reconstructions employing identical mode structure show improved quality (Figure 4) both for reconstructed object function as well as the illumination function. The illumination function resembles the eigen face of the dataset as it should. Unwanted structure corrupting the bright field has been eliminated.

**References**

[1] Kuppili, V. S. C. (2020). X-ray Far-Field Ptychotomography at I13-1 Diamond Light Source, Dissertation.

[2] Cowley, J. M. (1969). Image contrast in a transmission scanning electron microscope.Applied Physics Letters , 15(2):58–59.

[3] Cowley, J. M. (1992). Twenty forms of electron holography.Ultramicroscopy , 41(4):335–348.

[4] Pelz, P. M., Guizar-Sicairos, M., Thibault, P., Johnson, I., Holler, M., and Menzel, A. (2014). On-the-fly scans for X-ray ptychography.Applied Physics Letters ,105(251101):1–5.

[5]Clark, J. N., Huang, X., Harder, R. J., and Robinson, I. K. (2014). Dynamic imaging using ptychography. Physical Review Letters , 112(11):1–5.

[6]Huang, X., Lauer, K., Clark, J. N., Xu, W., Nazaretski, E., Harder, R., Robinson, I. K.,and Chu, Y. S. (2015). Fly-scan ptychography.Scientific Reports , 5(9074):1–5.

[7]Enders, B., Dierolf, M., Cloetens, P., Stockmar, M., Pfeiffer, F., and Thibault, P.

(2014). Ptychography with broad-bandwidth radiation. Applied Physics Letters, 104(17):1–5

[8] Jonathon Shlens (2014) arxiv:1404.1100v1.

[9]https://docs.scipy.org/doc/scipy/reference/generated/scipy.ndimage.gaussian_filter.html


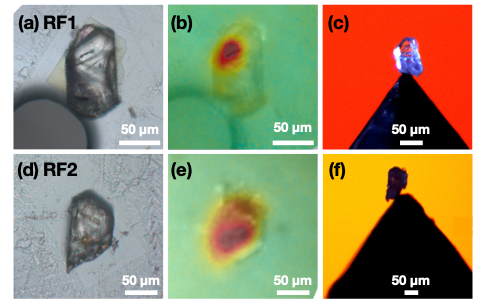


**Fig. S1**. (a, d) Optical microscopy images of selected single-crystal zircons RF1 and RF2 embedded in epoxy. Circular feature on lower left of (a) is an air bubble. (b, d) QDM map of the out-of-plane component of magnetic field manually registered by eye and overlain on top of the optical images. Samples were given a saturation isothermal remanent magnetization using an out-of-plane field prior to measurement. (c, f) Optical image of zircons mounted on sharp tungsten carbide pins using UV setting glue ready for X-ray ptycho-tomography measurements.


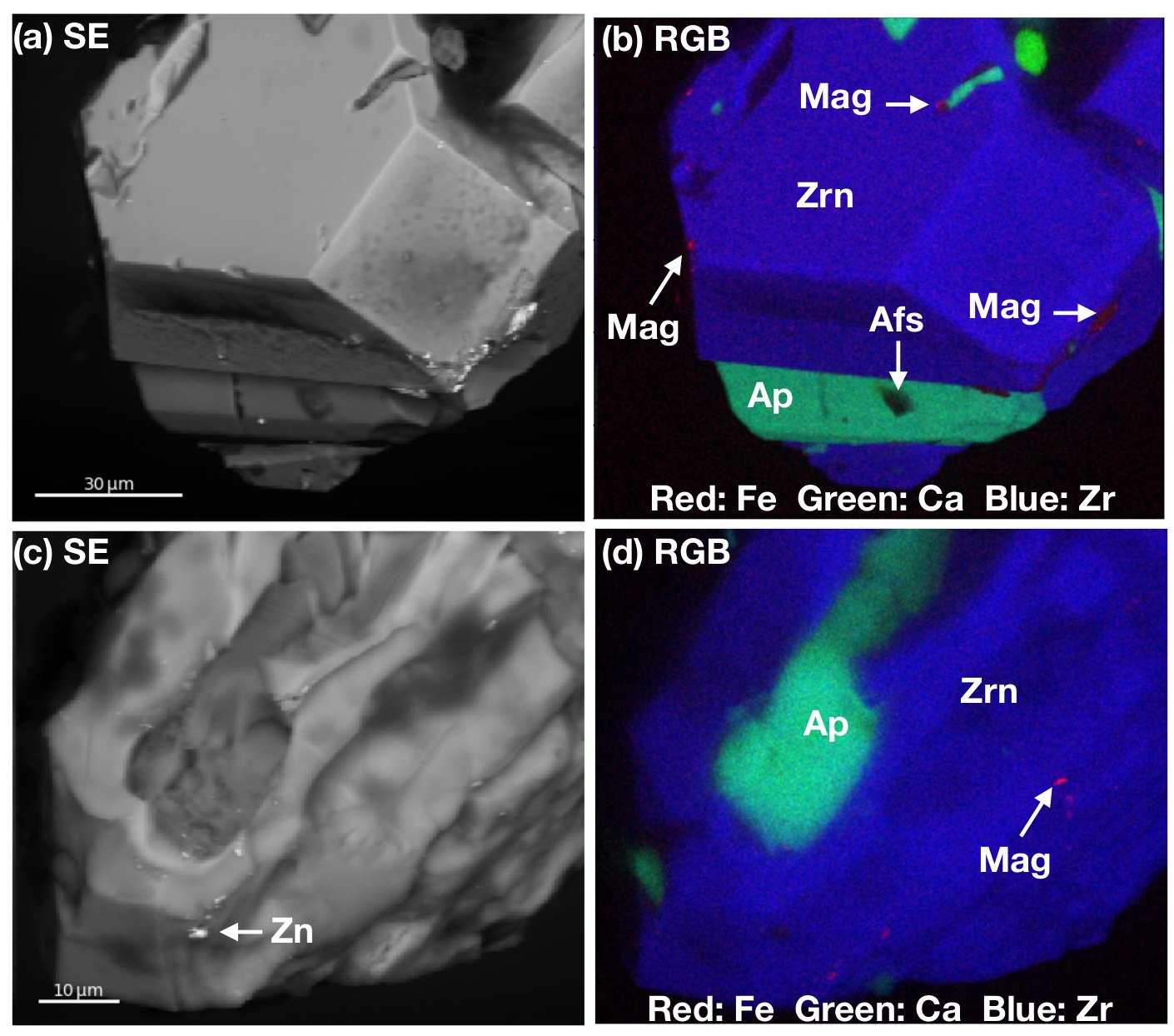


**Fig. S2**. (a, c) Secondary electron (SE) SEM images of samples RF1 and RF2, respectively. (b, d) RGB colour images formed by combining Fe Kα (red channel), Ca Kα (green channel) and Zr Lα (blue channel) EDX chemical maps of samples RF1 and RF2, respectively. Zrn = zircon, Ap = apatite, Mag = magnetite, Afs = alkali feldspar, Zn = zinc-rich surface contamination.


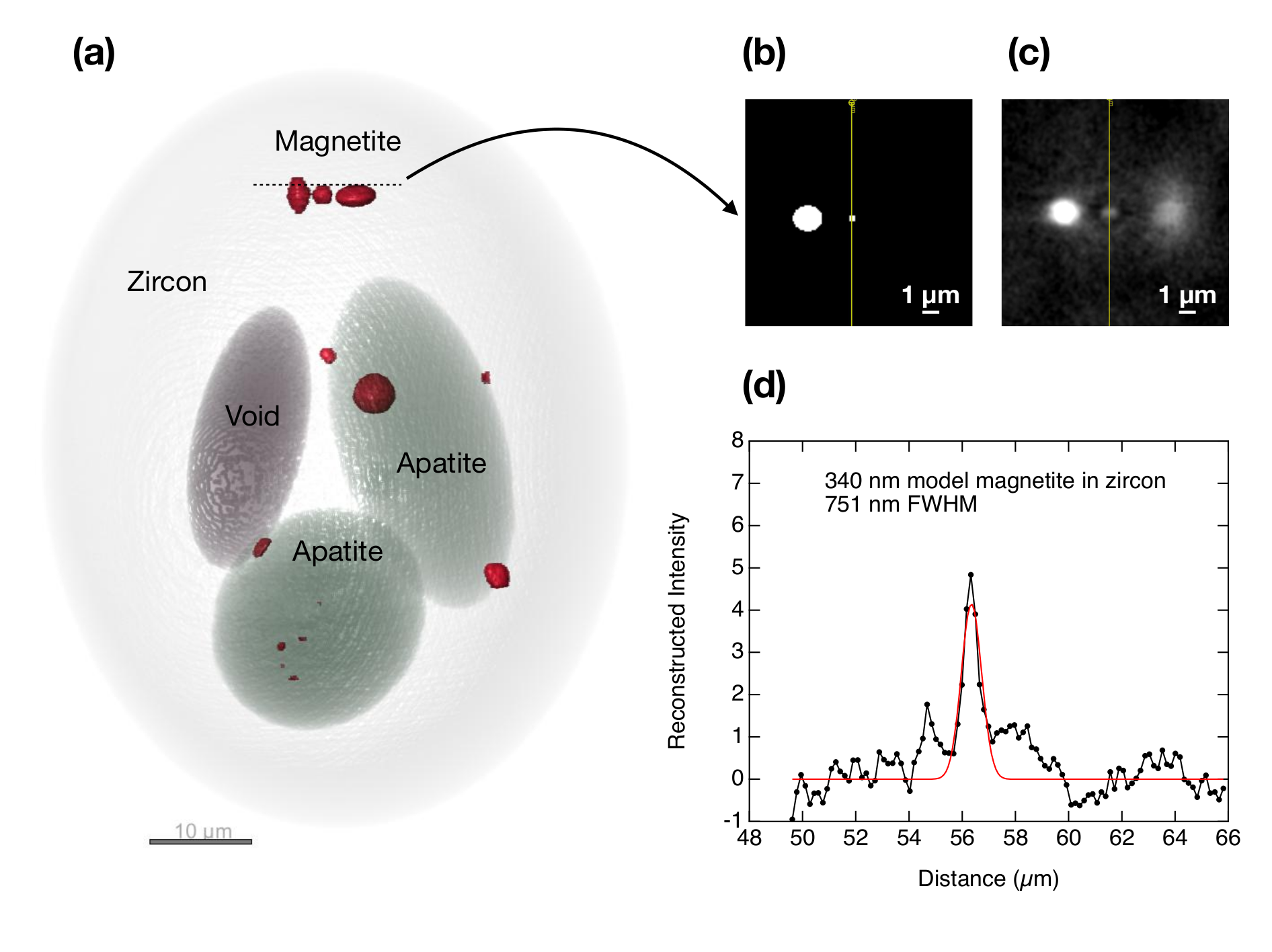


**Fig. S3**. (a) Internal reconstruction of ellipsoidal phantom zircon crystal (light grey) containing ellipsoidal inclusions of apatite (green-grey), void space (dark grey) and magnetite (red). Both the model structure and reconstruction are based on 85x85x85 nm voxel resolution. Reconstruction was performed using the same experimental geometry as those shown in Figs. 6 and 7. (b) 2D slice through the model structure taken at the level indicated by the dashed line in (a), which slices through the volume of the left-hand magnetite particle, through the top layer of the central magnetite particle, and one layer above the righ-hand particle. (c) Corresponding 2D slice through the reconstruction demonstrating both the broadening of the central particle and the bleed-through image of the right-hand particle.


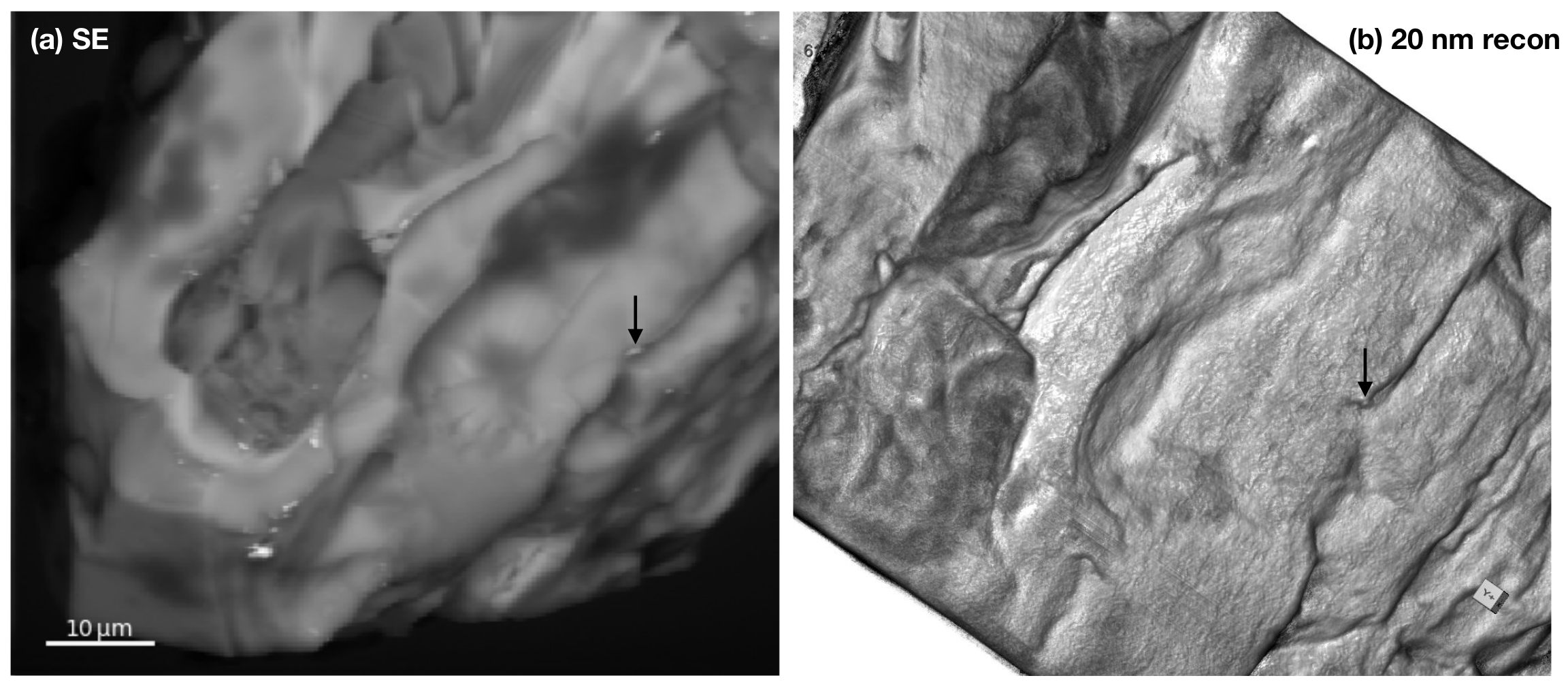


**Fig. S4.** (a) Secondary electron (SE) SEM images of sample RF2. Arrow highlights particle at surface that was identified as Fe-rich using EDS in Fig. 2d. (b) Partial surface reconstruction of RF2 using 20 nm resolution voxels. Black arrow highlights the same feature as in (a).

**Fig. S5.** Generated phantom comprising 6 magnetite spheres of diameters 5000, 1000, 500, 300, 200 and 100 nm arranged in a spiral fashion. The spheres are embedded midway in a 100 µm thick zircon matrix. (b) Shows the zoomed in picture contained in the red rectangle.

**Fig. S6.** Ptychographic reconstructions of the forward modeled data sets from Fig. S5. As expected, we see improved resolution with increase in flux (i.e., higher scaling factors) incident on the sample.

**Fig. S7.** Regions of interest from three different reconstructions at different reconstruction pixel sizes. The regions correspond to a single diffraction pattern in each case (Fig. S8). A 300-400 nm particle is resolved in 5nm reconstruction, but it is absent in the other two.

**Fig. S8.** Signal vs D-spacing plots of the diffraction frames corresponding to the regions of interest shown in Fig. S7.

**Fig. S9.** Figure showing ptychographic reconstructions from synthetically scaled experimental data sets and reconstructions (**SSED**). The particle of concern vanishes with a reduction in photons.

**Fig. S10.** Ptychographic reconstructions from forward modelled ptychographic data sets obtained from scaled illumination functions (**FMSIL**). We see similar effects as SSED in Fig. S9 where the particle gets obscured with a reduction in photons/scaling factor.

**Fig. S11.** Synthetic phantom comprising 7 magnetite spheres of diameters 5000, 1000, 500, 300, 200, 100 and 50 nm arranged in a spiral fashion. The spheres are embedded midway in a 100 µm thick zircon matrix. (b) Shows the zoomed in picture contained in the red rectangle. (c) Ptychographic reconstructions of the forward modeled data sets with a scale factor of 100. The 50 nm sphere is barely resolvable.


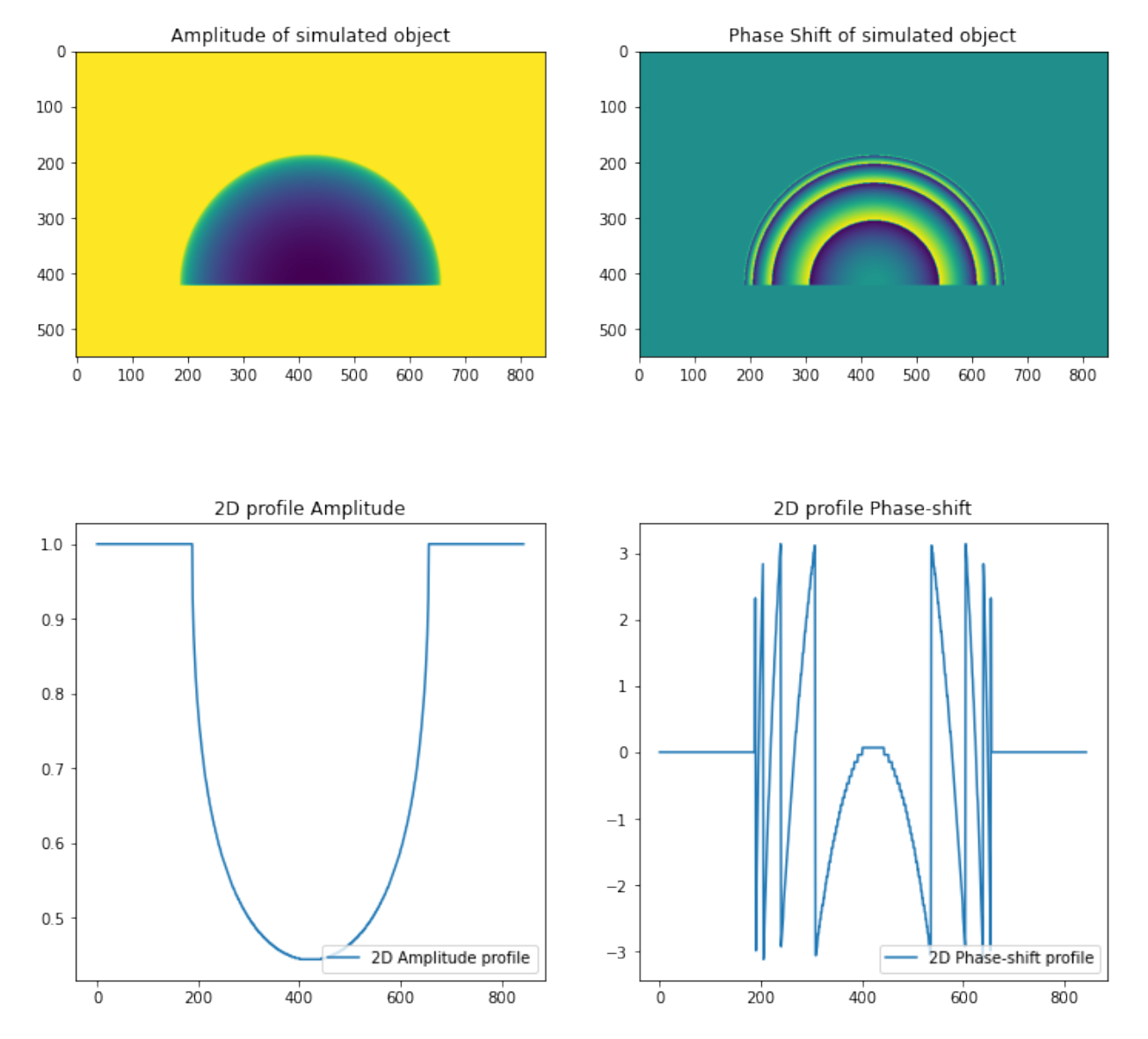


**Fig. S12**. Simulation of a thick sample containing multiple phase wraps. A 2D map of the amplitude of the simulated object is shown in the upper left. A 2D map of the corresponding phase-shift is shown in the upper right. The lower images show 1D profiles of amplitude and phase-shift of the same sample at the 400th row (red dotted line). While the amplitude never goes below (1/e th) value, the phase wraps multiple times.


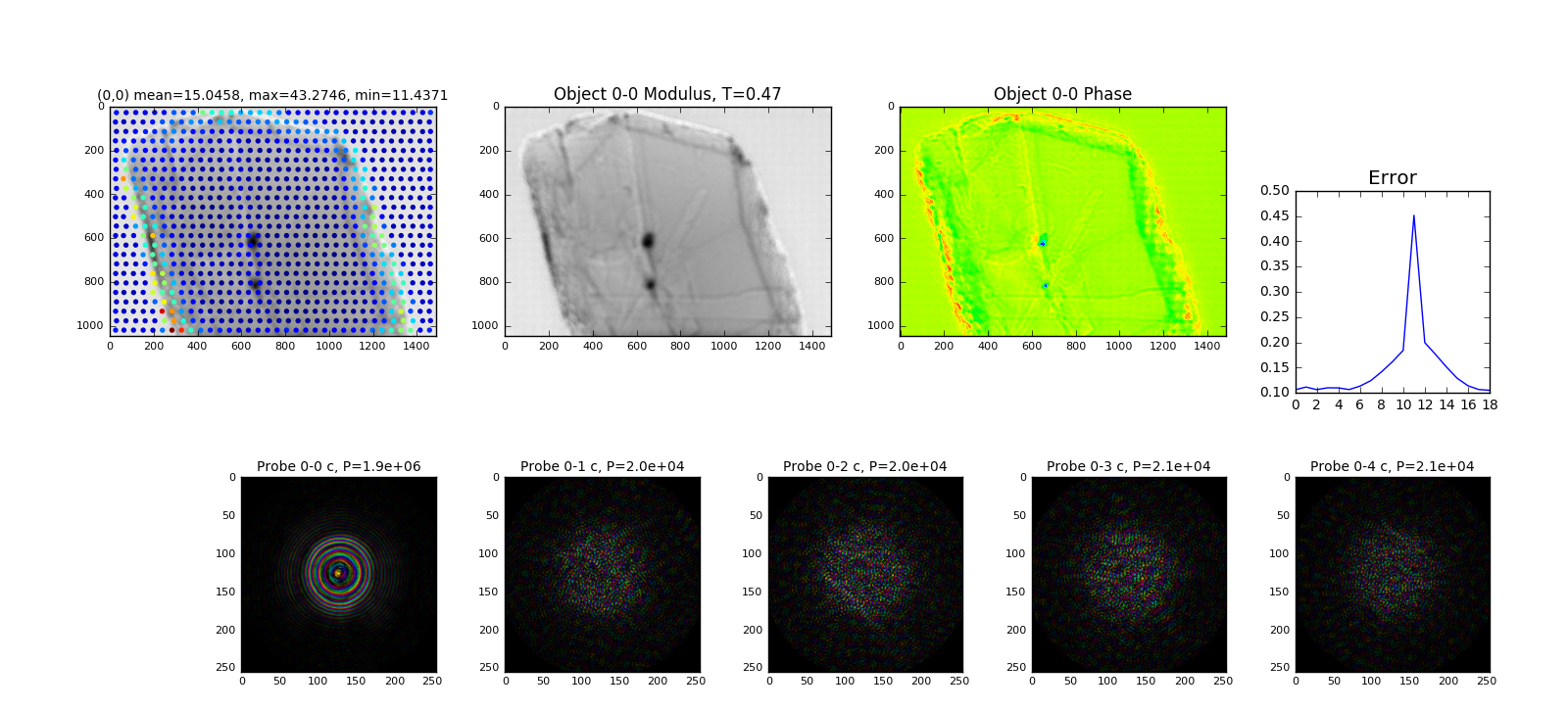
**Fig. S13**. Ptychographic reconstruction of thick 3D zircon samples at the 18th iteration step. The reconstruction process initially reconstructs the “edge” features within the transmission function. In the initial stages, phase-shift doesn’t depict the reality accurately.


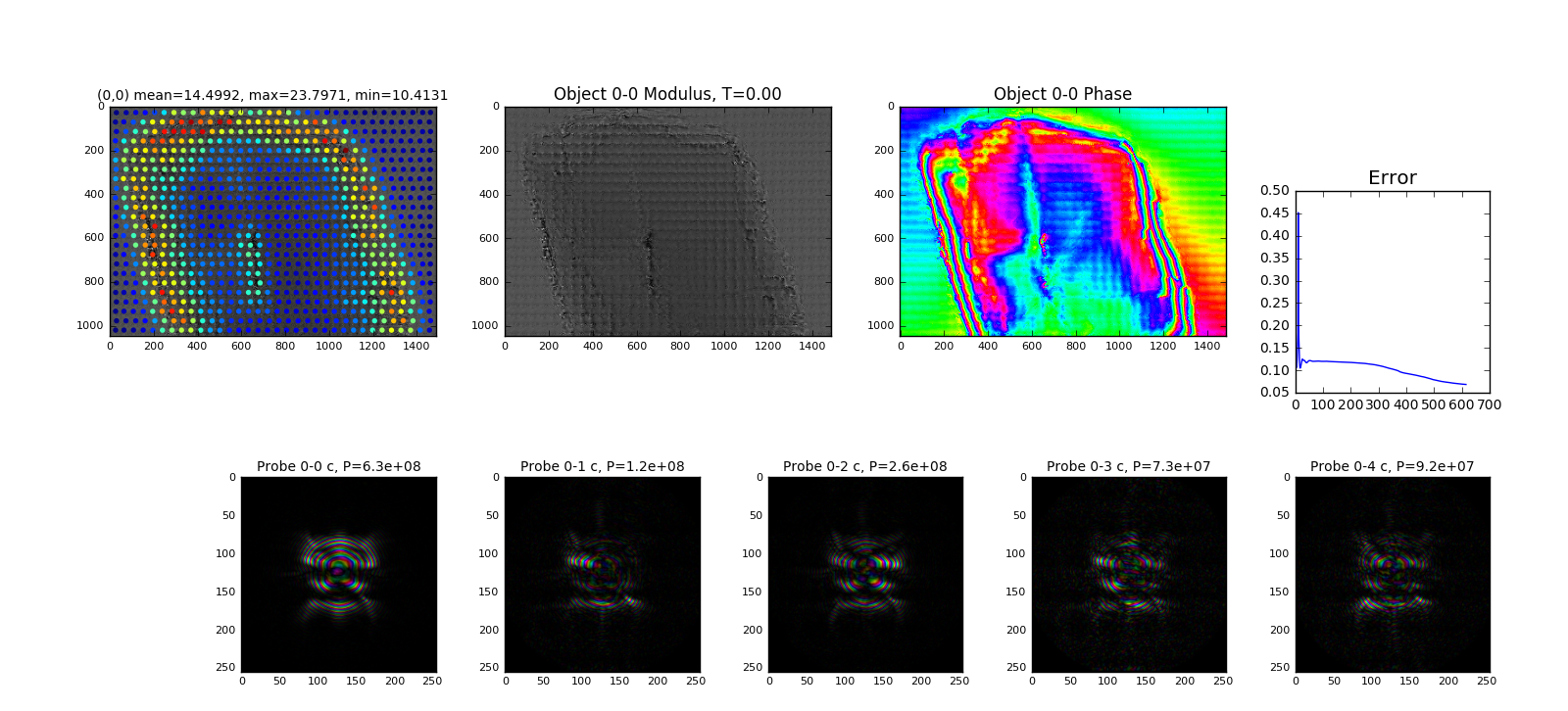


**Fig. S14**. Ptychographic reconstructions of thick 3D zircon samples at 615th iteration step. Over the course of the reconstruction process, the algorithm (rightly) starts reconstructing the phase wrapping aspect of the phase-shift of the object’s transmission function. The reconstruction process however introduces artifacts into the amplitude of the object’s transmission function.


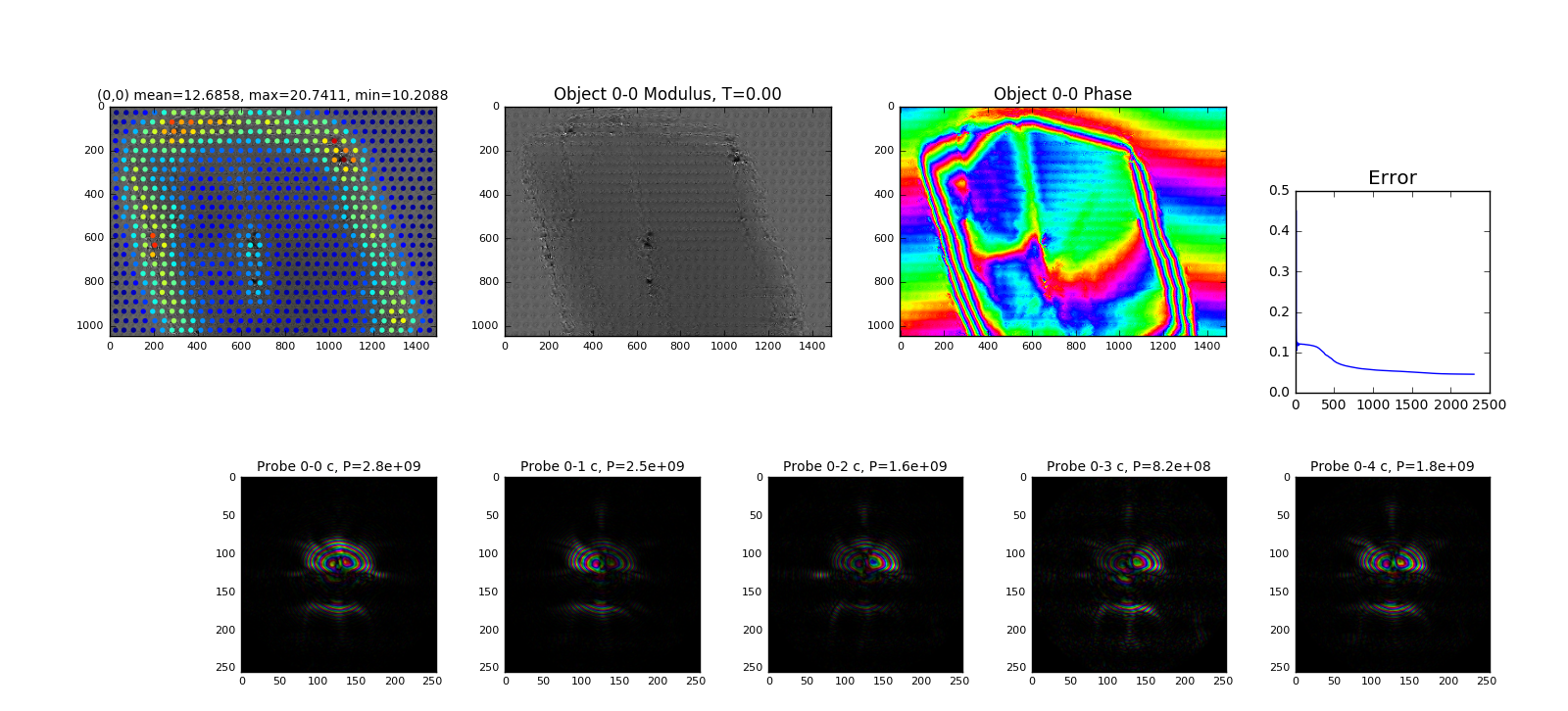
**Fig. S15**. Final state of the sub-optimal ptychographic reconstruction. One can see that while the algorithm rightly reconstructs the phase wrapping of the phase-shift of the object’s transmission function it still introduces pock-marked artifacts into the ptychographic reconstruction. The reconstructed probe also looks sub-optimal.


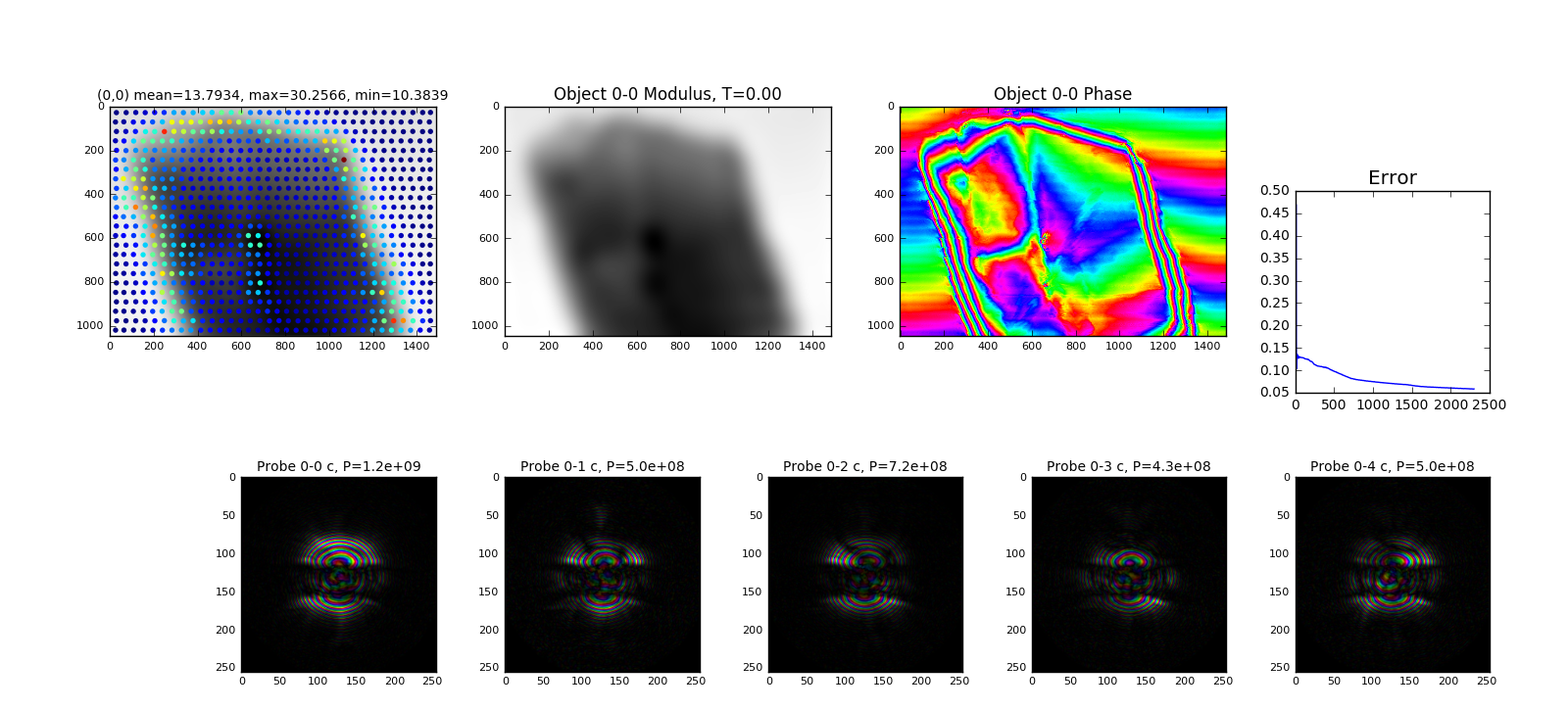
**Fig. S16**. Figure showing the final snap shot of the reconstruction while using the Gaussian blur step. One can see that the pock-marked artifacts in the reconstructed phase have now vanished. The reconstructed probe still looks sub-optimal, however.


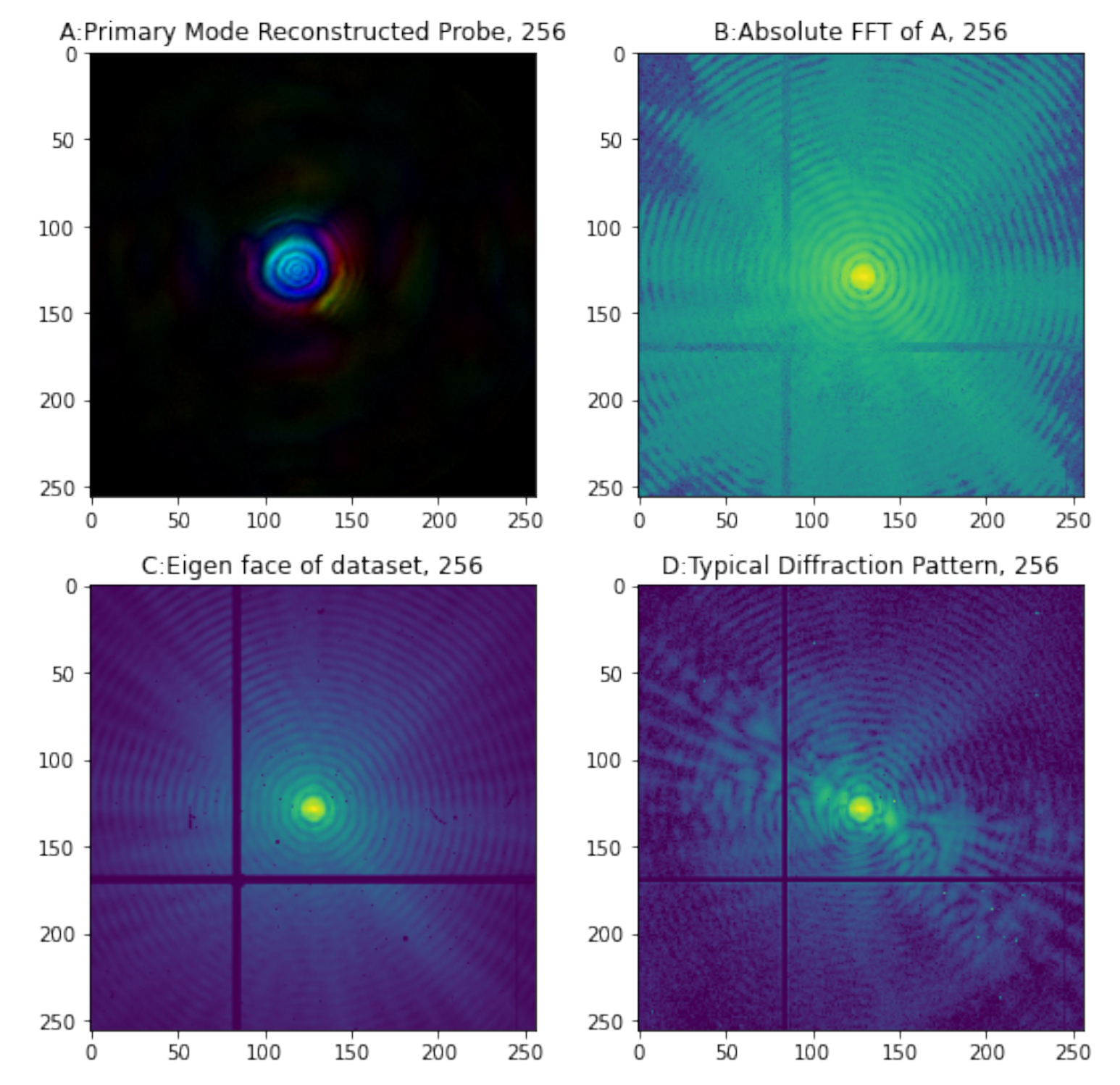


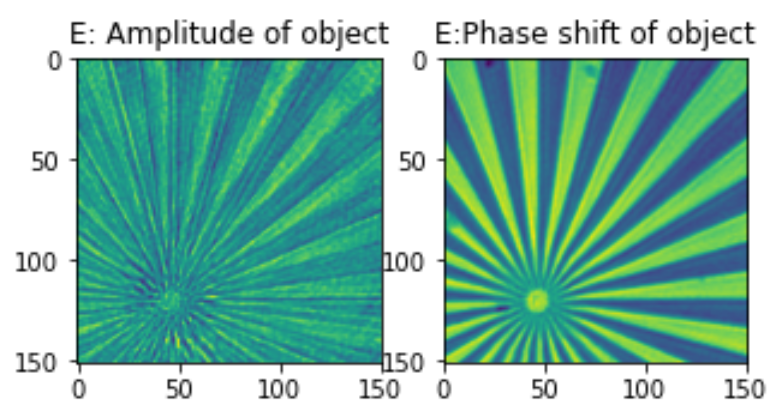


**Fig. S17**. A shows the reconstructed illumination function employing ptychography. B shows the amplitude of the Fourier transform of the function shown in A. The primary mode of the PCA of the dataset from which the reconstruction has been obtained is shown in C. A typical Far-Field diffraction pattern from the same dataset is shown in D. One can see that the illumination function retains all the position/time invariant features of the total dataset. E shows the object function of the ptychographic reconstruction.


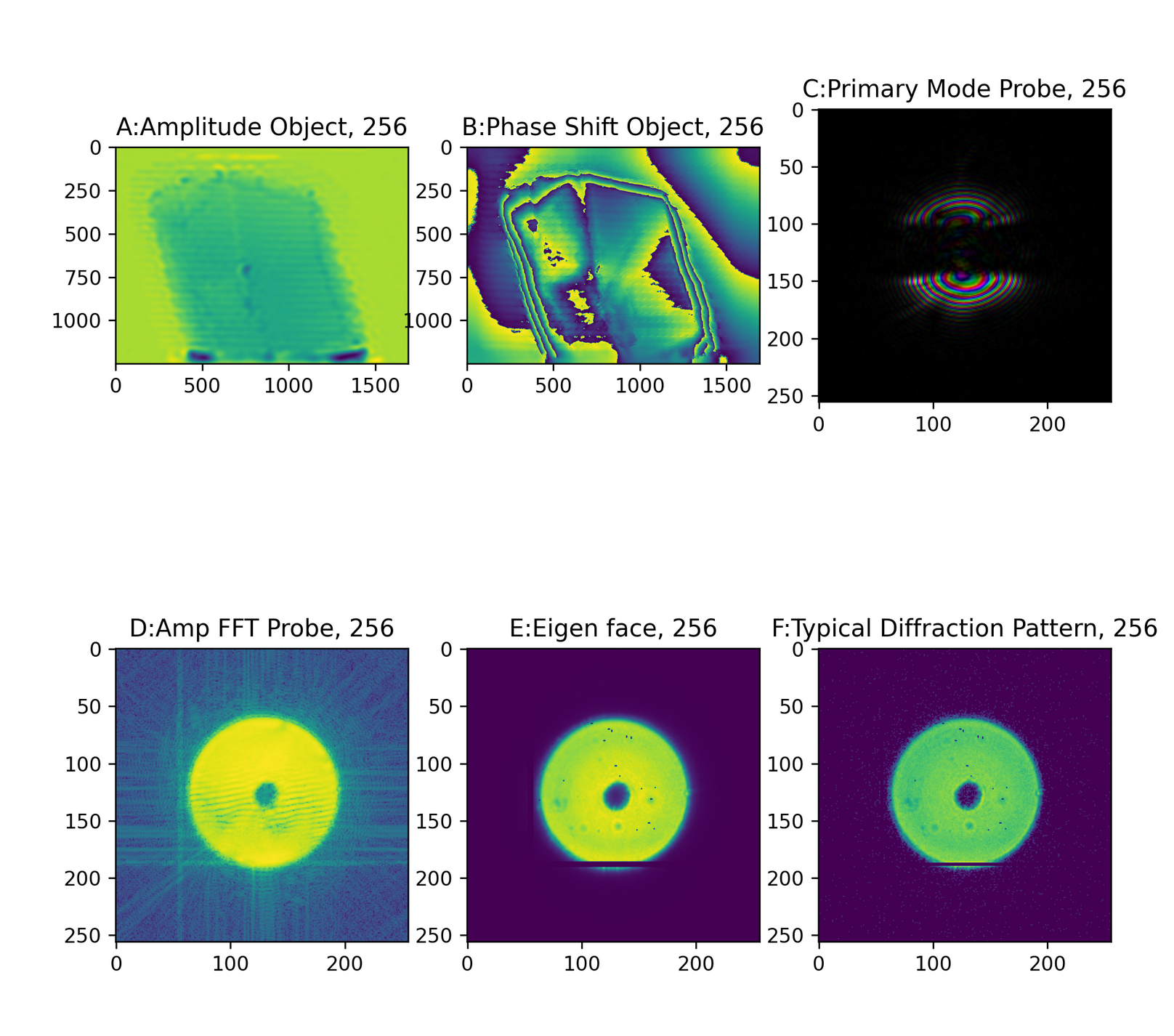
**Fig. S18**. A, B shows the amplitude, phase-shift of the reconstructed object through ptychographic reconstruction. C shows the reconstructed probe function. Both from the object and probe, one can clearly see that reconstruction is sub-optimal. Using the new tool introduced in this study, one can see the issue with the reconstruction namely unwanted structure in the bright-field part of the probe (D). Comparing the same with the eigen face of the dataset (E) along with a typical diffraction frame (F), one can see that the unwanted structure could be the reason behind the sub-optimal results.


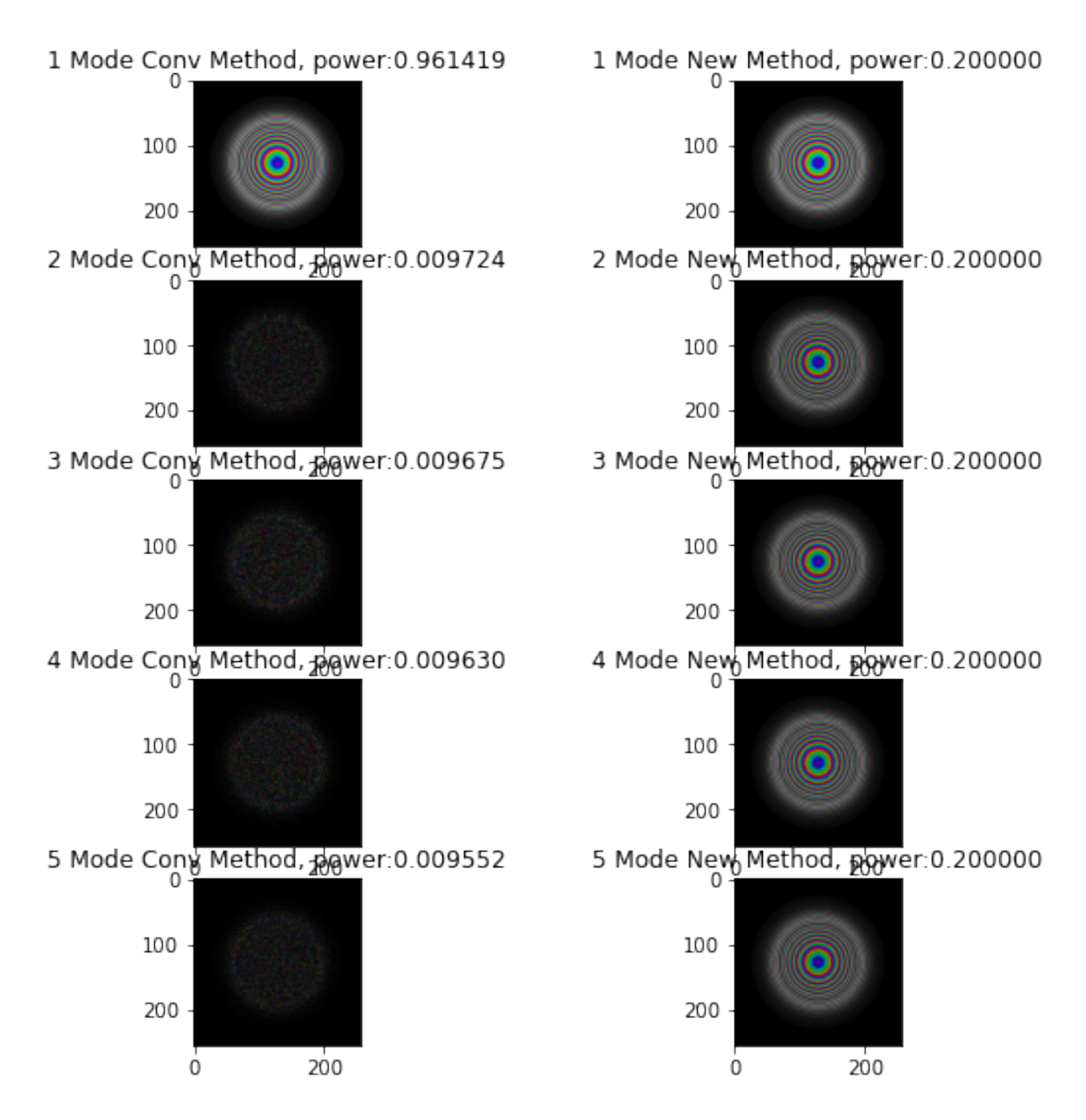
**Fig. S19**. Initial probe guess for the conventional method vs the new method. In the conventional method, primary mode contains the illumination function (pin hole or zone plate) function with majority of power. Rest of the modes contain noise with nominal power. New method contains identical illumination function with identical power distribution for all modes.


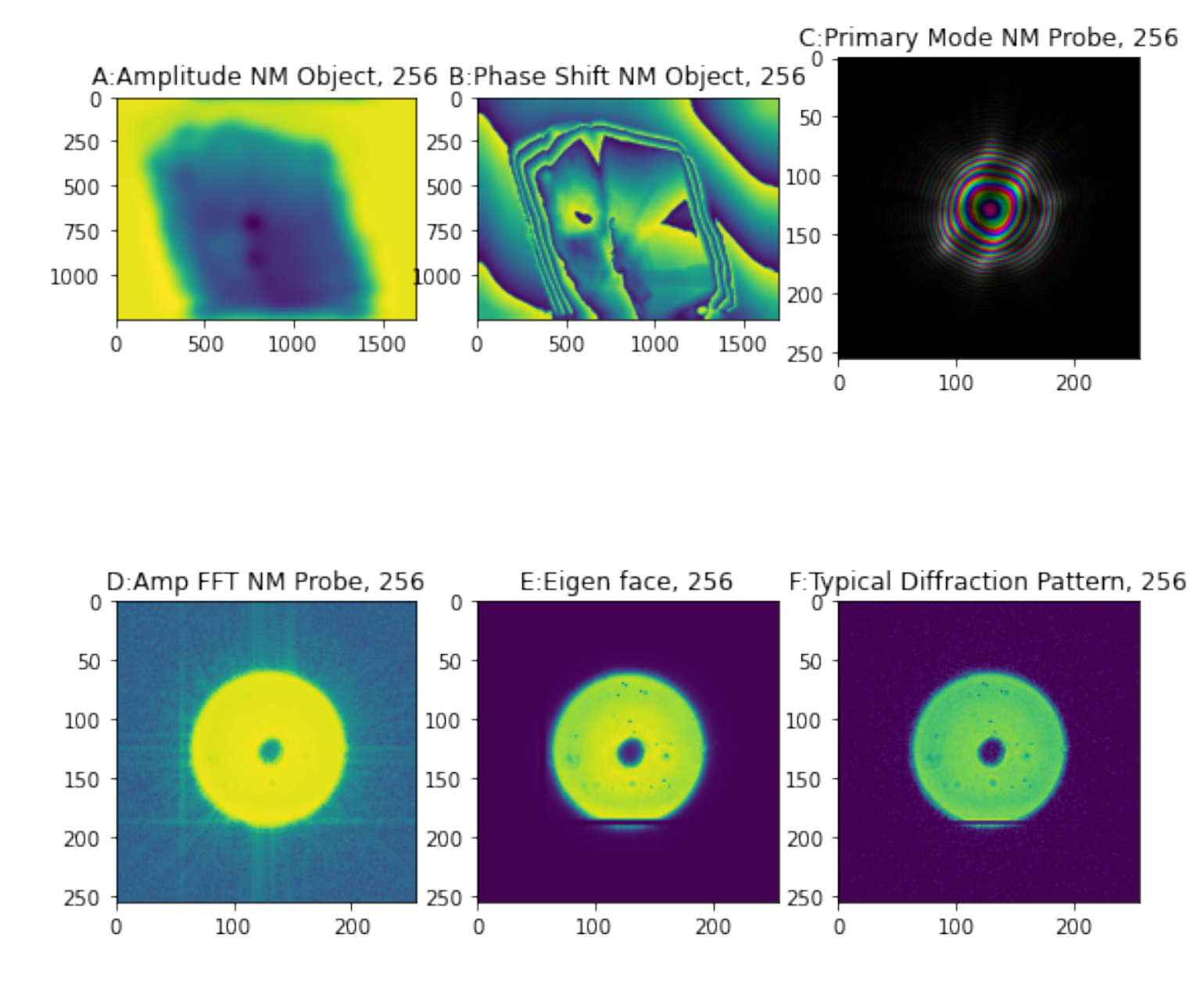
**Fig. S20**. A, B shows the amplitude, phase-shift of the reconstructed object through new method. C shows the reconstructed probe function through the new method. Both from the object and probe, one can clearly see that reconstruction has been improved immensely. Using the new tool introduced in this study, one can see that the probe’s bright field (D) no longer contains the unwanted structure along with this, the Fourier transform of the reconstructed probe (D) looks similar to the eigen face of the dataset (E). F shows a typical diffraction pattern from the same dataset.


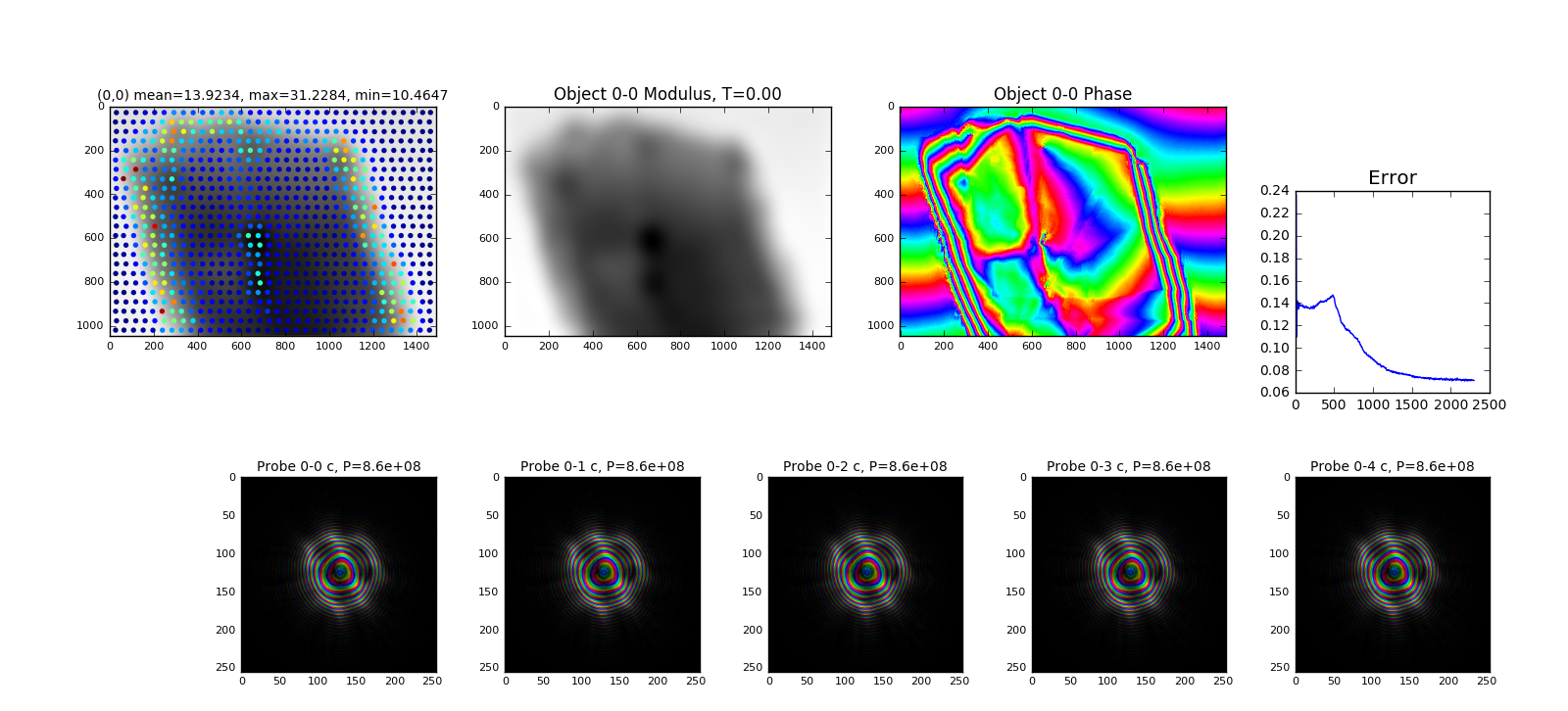


**Fig. S21**. Figure showing the optimum ptychographic reconstruction after dealing with multiple challenges involving ptychography of dense 3D samples.


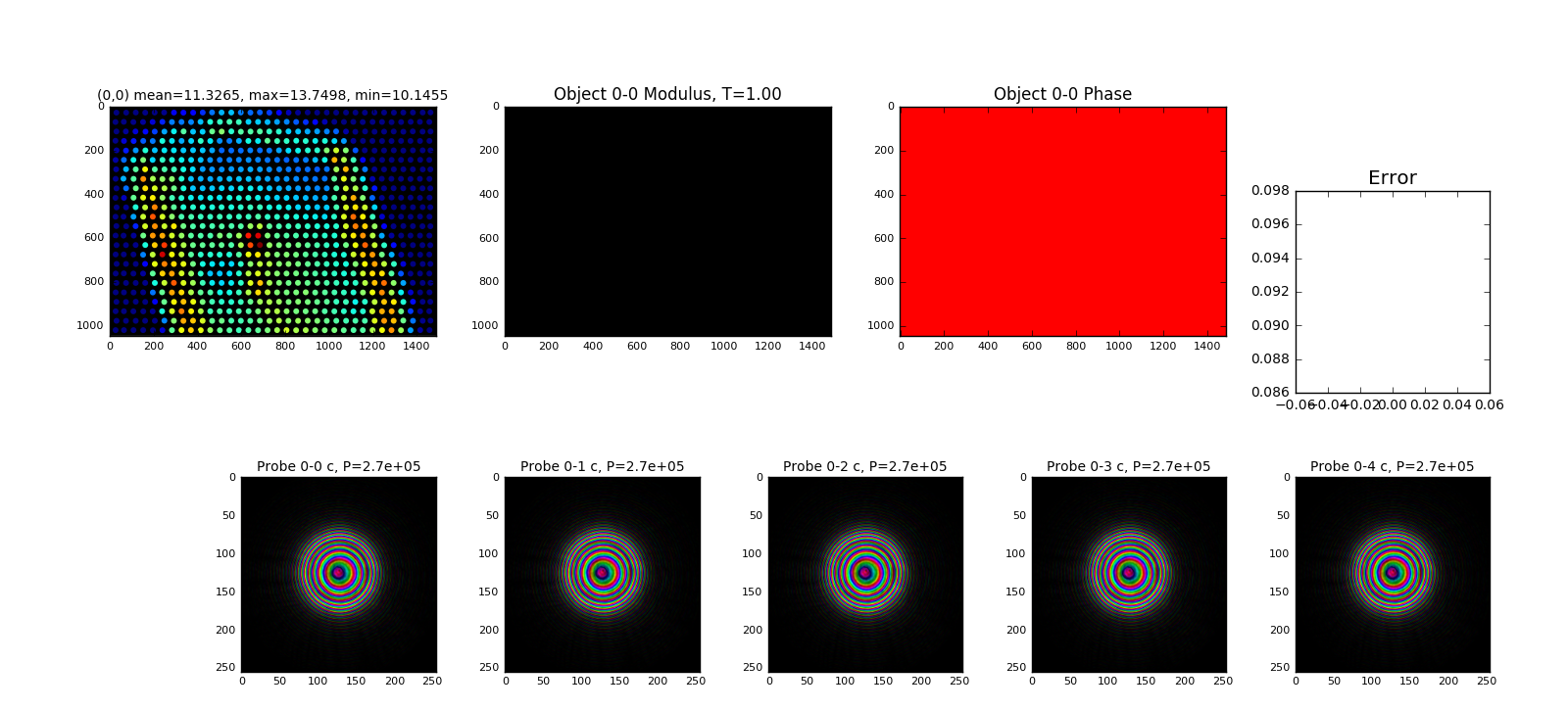


**Fig. S22**. Figure showing the state of Fig. S21 at first iteration, probe function was borrowed from a different reconstruction and was employed with identical mode structure. Object was assumed to be an array containing 1s.
